# Morphological and taxonomic descriptions of a new genus and species of killifishes (Teleostei: Cyprinodontiformes) from the high Andes of northern Chile

**DOI:** 10.1371/journal.pone.0181989

**Published:** 2017-08-08

**Authors:** Gloria Arratia, Irma Vila, Natalia Lam, Claudia Jimena Guerrero, Claudio Quezada-Romegialli

**Affiliations:** 1 Biodiversity Institute, University of Kansas, Lawrence, Kansas, United States of America; 2 Departamento de Ciencias Ecológicas, Facultad de Ciencias, Universidad de Chile, Santiago, Chile; 3 Departamento de Producción Animal, Facultad de Ciencias Agronómicas, Universidad de Chile, Santiago, Chile; 4 Escuela de Medicina Veterinaria, Universidad Iberoamericana Ciencia y Tecnología, Santiago, Chile; 5 Instituto de Ciencias Naturales Alexander von Humboldt, Universidad de Antofagasta, Antofagasta, Chile; Universiteit Gent, BELGIUM

## Abstract

A new genus and species, *Pseudorestias lirimensis*, is described from the southern part of the Chilean Altiplano. While sharing several characters that clearly align the new species with *Orestias*, this new fish is characterized by numerous autapomorphies: the Meckel cartilage is a continuous cartilage that broadly expands posteriorly (in large specimens, it keeps its anterior part and is resorbed posteriorly), the basibranchials are fused into one long element, the second pharyngobranchial is not displaced dorsally over pharyngobranchial tooth plate 3+4, but they are aligned, the anterior and posterior ceratohyals are closely articulated keeping a scarce amount of cartilage between both bones and ventral to them, ossified middle and distal dorsal radials are present in females as well as ossified middle and distal anal radials. *Pseudorestias lirimensis* presents strong sexual dimorphism associated to size. Females are almost twice as large and long than males, neuromast lines are absent in males, a mesethmoid is present in males, squamation on head is reduced in males, and ossified middle and distal radial of dorsal fin are cartilaginous in males. *Pseudorestias* and *Orestias* are suggested as the sole members of the tribe Orestiini. A list of characters diagnosing the tribe is provided. The presence of the new genus is interpreted as a possible result of the ecosystem isolation where the fish is living from surrounding basins—as early as possibly from the Miocene-Pliocene times—and its physical and chemical characteristics. Small populations, living conditions, small habitat, and reduced distribution make this species a strong candidate to be considered critically endangered, a situation already established for all other Chilean species living in the Altiplano. There is high probability it will become extinct due to water demands and climate change in the region.

## Introduction

Cyprinodontiformes, or killifishes, are known by an approximate number of 1,260 species, which are included in ten families [1] of primarily living in freshwater ecosystems. The family Cyprinodontidae includes approximately 130 species. Members of the subfamily Cyprinodontinae are classified in two tribes: the Cyprinodontiini and the Orestiini [1–4], but their contents and phylogenetic relationships are in dispute (see [2,3] versus [4]). Nevertheless, current literature [1] follows Parenti [2,3] after a reevaluation of previous studies on atherinomorphs by Parenti [5], which confirmed her previous results [2,3]. Orestiasini (= Orestiini) was erected by Bleeker in 1859 [6] to contain only species of *Orestias*, an interpretation also reached by Costa in 1997 [4]. Consequently, Orestiini *sensu* Costa [4] would be restricted to the Altiplano of South America. In contrast, Orestiini *sensu* Parenti [2,3] would include two extant genera, *Aphanius* and *Orestias*, which have different geographic distributions. *Aphanius* inhabits the Mediterranean region and the Mediterranean islands, as well as the Saudi Arabian Peninsula and Iran in the Northern Hemisphere (e.g. [7,8]). *Orestias* inhabits the Altiplano of Bolivia, Peru, and Chile (e.g., [3,9]) along the Continental Divide of South America in the Southern Hemisphere. All other South American cyprinodontiforms inhabit the Neotropical Region of the continent (e.g. [2,10–12]). In addressing the geographical distribution of the fishes, the Eurasian and American disjoint occurrence of the freshwater members of the tribe Orestiini is considered a major biogeographic challenge to Parenti’s [2] hypothesis of relationships based solely on morphological characters (for different biogeographic interpretations on the distribution of *Orestias* versus *Aphanius* see [4,13]). Parker and Kornfield [14], however, corroborated the close relationship of *Orestias* with the Anatolian cyprinodontids in a molecular study and proposed that the tribe Orestiini represents a Tethyan distribution pattern. Nevertheless, a recently published molecular study [15], which did not include *Orestias*, reached different results—with Cyprinodontidae not monophyletic and *Aphanius* and *Valencia* as sister taxa.

**Fig 1:**
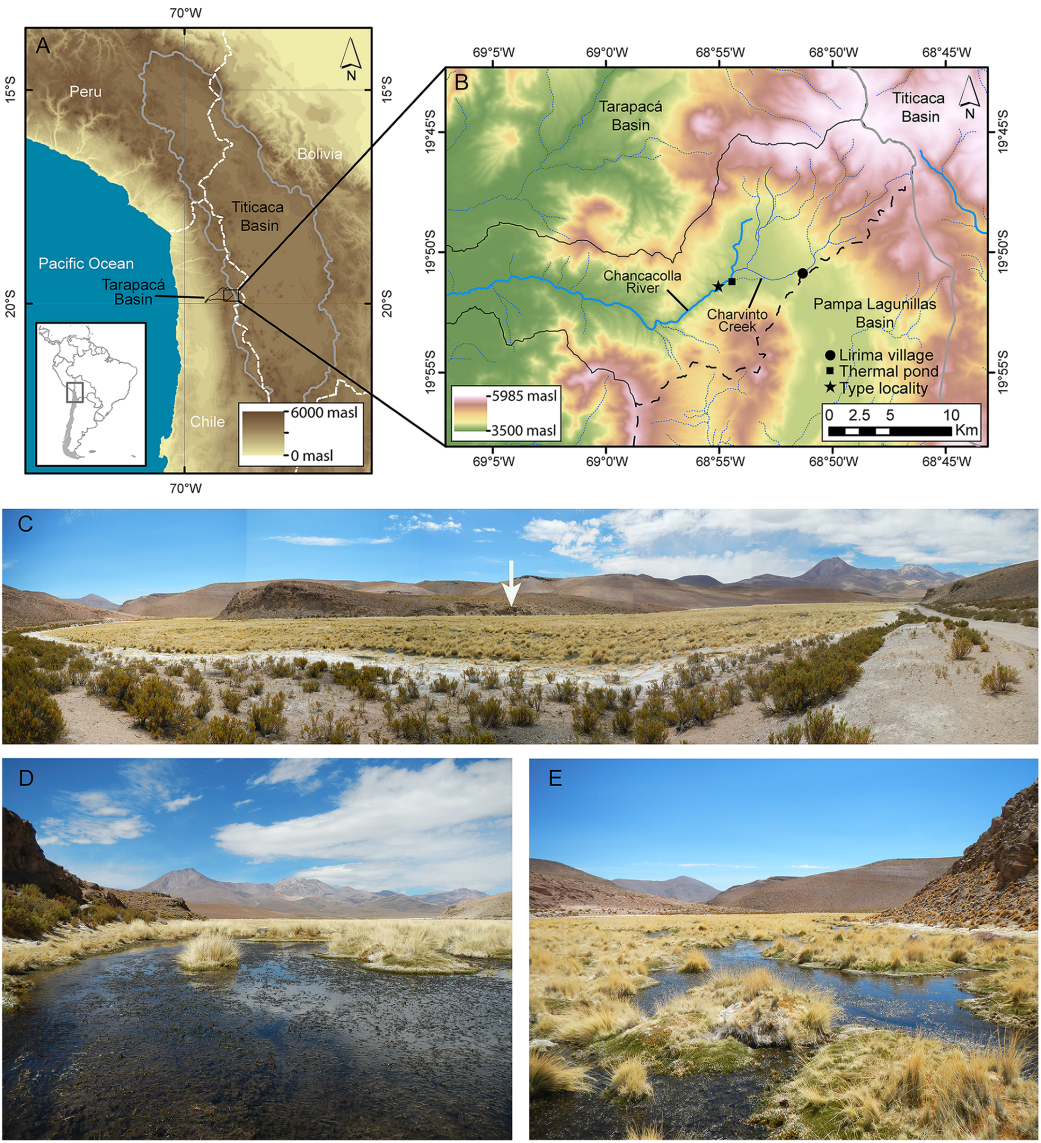
Geographical placement of the locality containing *Pseudorestias lirimensis* gen. et sp. nov. **A**, General overview of High Andean systems in South America. **B**, Detail of Chancacolla River and its streams and with the type locality indicated [with a star]. **C**, Panoramic view of the Chancacolla River in the type locality. The arrow points at **D** main course of Chancacolla River facing upstream, and (**E**) facing downstream and showing characteristic association of macrophytes and vegetation that comprises a wetland (= bofedal). C, D and E are photographs by C. Quezada-Romegialli, whereas A and B are figures based on Shuttle Radar Topography Mission (SRTM GL1) Global 30m [40] provided by the OpenTopography Facility (see conditions of use in S2 Text) with support from the National Science Foundation under NSF Award Numbers 1226353 & 1225810 (https://doi.org/10.5069/G9445JDF). Identifications of rivers and Lirima village are from CQR.

The Andean Altiplano (Fig 1A), a wide plateau at 3 km elevation between 13 and 27° S [16] contains a large, closed drainage basin between the East and the West Andes ranges. This is the Altiplano Basin (*sensu* [16]) or the Titicaca Ecoregion (*sensu* [17]). This plateau is located between Peru, Bolivia, Argentina, and Chile respectively. It reaches elevations between 3,000 to over 5,500 m above sea level (m a.s.l.). This region is well-known by the Desaguadero River which is formed at the exit of the Titicaca Lake (about 3,800 m a.s.l.) and connects with the Poopó Lake (about 3,600 m a.s.l.) and sporadically with the Uyuni Saltpan (at 3,600 m a.s.l.)—the largest saltpan in the world. This region also corresponds to the northeast part of the Altiplano. In contrast to the northern Altiplano, the Chilean or southern Altiplano holds smaller water bodies such as Chungará Lake (at 4,517 m a.s.l.), one of the highest lakes containing fishes in the world [18,19], and numerous small rivers, “bofedales” or wetlands, and saltpans [20].

Members of the tribe Orestiini [*sensu* 1–3] consisting of approximately 80 species are currently classified in two genera: *Aphanius* (= synonyms *Kosswigichthys* and *Anatolichthys*) and *Orestias* [3,21–25]. In contrast, Orestiinii *sensu* [4] would contain about 47 species of *Orestias* (the counts includes the recently described species from the Chilean Altiplano; see S1 Text). It has been hypothesized that members of the genus *Orestias* have inhabited a special region of the High Andes freshwater systems since Miocene times [26]. Today, the 47 species of *Orestias* remain distributed in a region of the Altiplano situated within the Ancash Province of northern Peru at 10° S (*O*. *elegans*; [3]) to the El Loa Province in Chile at 22° S (*O*. *ascotanensis*; [3,27]). As far as is currently known, six species of *Orestias* are endemic to the Chilean Altiplano (Suppl. 1). Some of these Chilean populations have been assigned to *O*. *agassii* (e.g., [28–30]), however, recent genetic and morphological studies suggest that the *Orestias* species are well-differentiated morphologically and genetically from *Orestias agassii* of the Northern Altiplano [26,31].

In the last few years of field work, small populations of fishes—interpreted preliminarily as *Orestias* cf. *agassii* [32]—were discovered near the Lirima village. This is located at about 4,000 m a.s.l. near the Chancacolla River, in the Tarapacá Region in the southern Chilean Altiplano. Morphological studies of the fishes have revealed vast differences with species of *Orestias*. Here we will describe a new genus and species of the tribe *Orestiini* inhabiting a restricted and isolated geographic area in the Chilean Altiplano. Contributions to the identification and understanding of its external and internal morphological features, coloration, and karyotype will be addressed together with new interpretation of the tribe Orestiini.

### Aquatic ecosystem

The Chilean Altiplano, placed between 17° S (Caquena River) and 27° S (Negro Francisco Lake) (*sensu* [16]) contains ca. 50 closed hydrographic freshwater basins with significant variations in their physicochemical (e.g., ionic concentrations) and geomorphologic characteristics [20,33]. Ongoing climate changes [32,34] and solar radiation in the area can be up to 20% greater than that recorded at sea level and at the same latitude [35]. Values of up to 1500 W/m^2^ [36] are easily reached. The majority of the aquatic ecosystems across this area remains unexplored and their diversity undescribed. This has limited the ability to understand their ecological importance and sensitivity to human impacts [32]. This shortcoming has been partially corrected by ongoing research conducted in the Chilean Altiplano during the last 40 years. These studies aim to characterize and describe the different basins and ecological composition in this area, e.g., Chungará Lake, Cotacotani and Piacota lagoons, wetlands and Lauca river (e.g., [23,26,31,32,37]).

One of these freshwater ecosystems, the Chancacolla River and its streams (Fig 1B), is the location where the new fish described here lives. Their side streams are considered wetlands situated at ca. 4,000 m a.s.l. in the Andes Range of the Quebrada de Tarapacá or so-called Taparacá Basin. The Chancacolla River is seasonally connected to other western rivers of the Taparacá Basin depending on the water flow. Other similar and closely located Chilean Altiplanic ecosystems receiving their water from groundwater, snow melts, and limited seasonal rains, possess a negative hydrological balance [32,35,38,39]. Thus, the Chancacolla River and its streams, supposedly possess a negative hydrological balance as well.

The Chancacolla River and surrounding wetlands in the type locality (Fig 1C–1E) has characteristic vegetation occurring alongside and laterally to the stream (Fig 1C). Cushion plants of the Juncaceae *Oxychloe* sp. and *Distichia* sp., and the Poaceae *Deyeuxia* sp. dominate the lawn communities. They are in a flatland of about 100–250 m wide and next to the river. Pools of variable diameter and depth form and these can range from a few to up to 50 cm depth. These environments constitute a special type of wetlands controlled by water quantity depending on seasonal water availability, favorable ambient temperature, and the availability of nutrients and biotic factors [41]. In this area, it is where the smallest individuals of the new species are commonly found. The stream itself (Fig 1D–1E) is shallow with less than 50 cm deep and up to 3 meters wide. It is seasonally covered by the Haloragaceae *Myriophyllium* sp. and the Apiaceae *Lilaeopsis* sp. Adults and bigger individuals were captured in the stream. The ionic content classifies the water of the Chancacolla River as a predominant sodium sulfated wetland (Na<Ca<K<Mg). See Scott et al.: table 1 [32] for physical and chemical variables.

## Material and methods

Considering the endangered status of Orestiini in the Chilean Altiplano and official restrictions concerning their fishing, only a total of 26 specimens were captured with hand nets and electro-fishing equipment directly from the Chancacolla River. A number of them were kept alive for photographing and chromosomal research. All specimens were euthanized using 500 mg/L tricaine methanosulfonate [42] and fixed in ethanol 70%, approved by the Bioethical Committee of the University of Chile. All specimens were collected with the permissions of the Subsecretaría de Pesca, Chile, under Exempted resolutions # 2231 of August 19, 2011 and # 1103 of April 24, 2015.

Sex identification was performed post-morten by examination of the gonads. Morphometric and meristic data for 20 specimens (see S1 Fig, S1 Table) were obtained following the methodology previously used to describe species of *Orestias* [3,43,44]. All measurements were based on straight-line distances and recorded with metric dial calipers of a reading to the nearest 0.1 mm. Cleared-and-stained specimens were prepared for cartilage and bone identification following established methodologies [45,46]. The branchial system was dissected and the gill rakers of the left gill arches 1 to 4 were counted in five specimens. Drawings were done by GA with Wild FM 8 and Leica MZ9 stereomicroscopes equipped with camera lucida attachments. Photographs of specimens and of the landscape were done by CQ-R. Chromosomal analyses were done by NL.

Chromosomal number and morphology was studied in five specimens. Mitotic chromosomes were obtained from squash preparations of gill epithelium following the procedures described in Vila et al. [23]. Twenty metaphase spreads from three females and two males were analyzed to confirm the diploid number and the karyotype structure. The chromosomes were classified as metacentric, submetacentric, subtelocentric, and telocentric according to Levan et al. [47]. The images were captured by digital camera CCD cooler (Nikon D60) in a Nikon Optiphot microscope. The karyotype mounting and adjustment of image brightness and contrast was carried out in Adobe Photoshop CS6.

Institutional abbreviations are listed in Sabaj Perez [48].

### Anatomical terminology

Most used morphological names follow Parenti [3] with the following exceptions: The terminology of fin rays follows Arratia [49] and that of the caudal skeleton follows Schultze and Arratia [50]. The terminology of the cephalic neuromast system follows Arratia and Huaquín [51] and it is explained herein.

**Fig 2:**
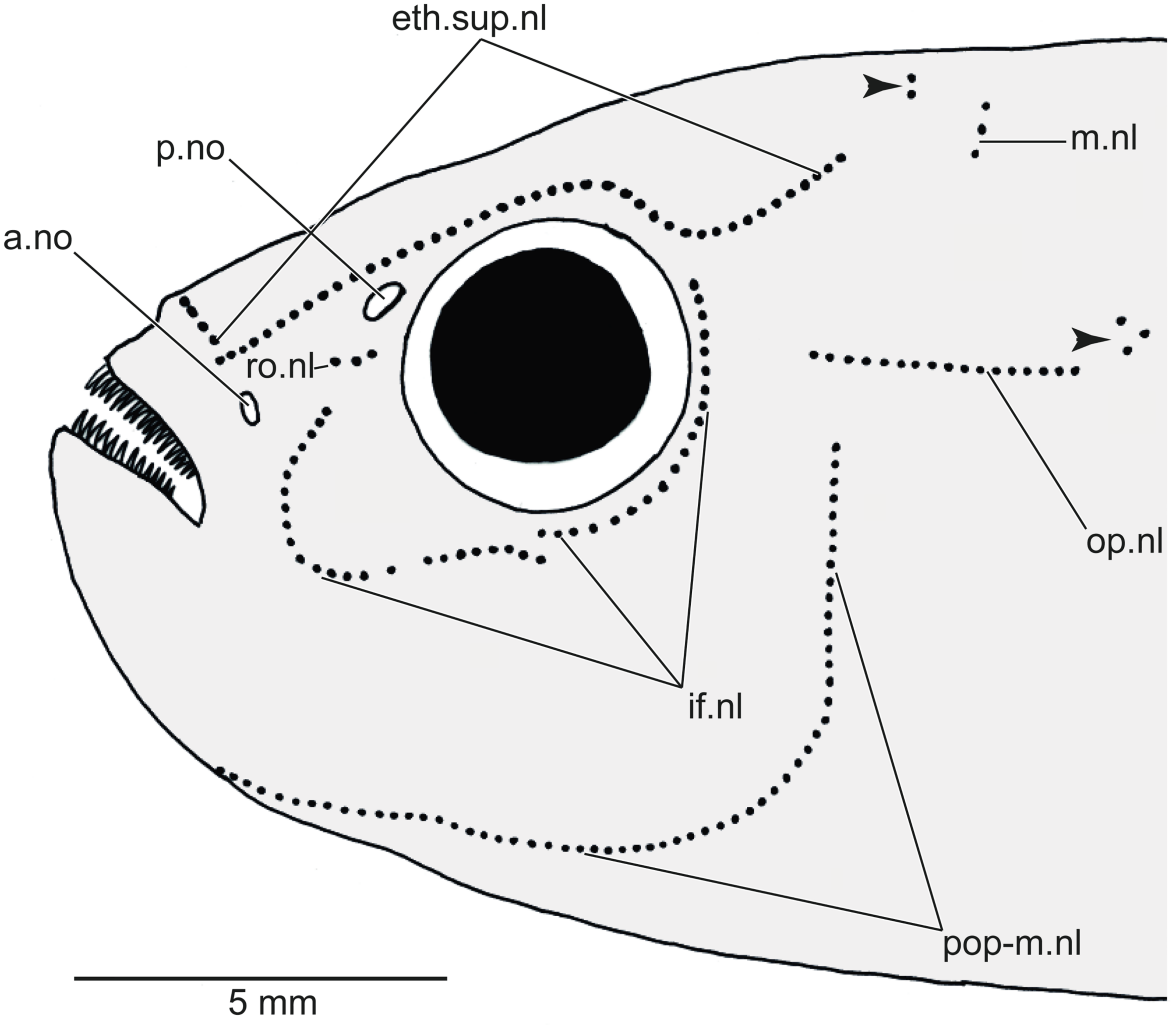
Diagrammatic representation of the neuromast lines and fields in orestiinids. Abb.: **a.no**, anterior nostril; **eth.sup.nl**, ethmoid-supraorbital neuromast line; **if.nl**, infraorbital neuromast line; **m.nl**, middle neuromast line; **op.nl**, opercular line; **p.no**, posterior nostril; **pop-m.nl**, preopercular-mandibular line; **ro.nl**, rostral neuromast line. Small arrowheads point to small fields of neuromasts.

**Fig 3:**
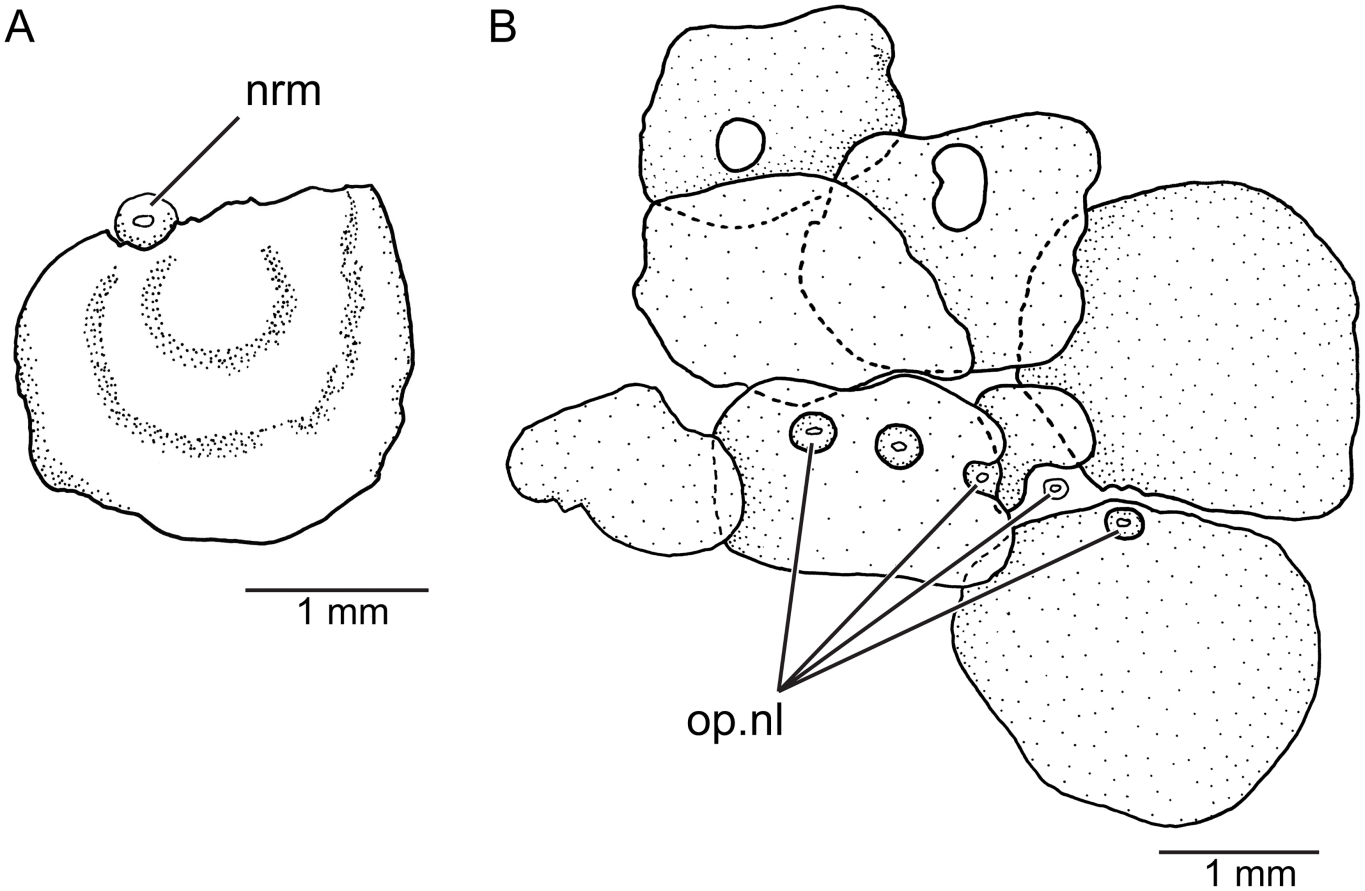
Neuromasts and their association to pitted scales in lateral views (KUNHM 41384, female). **A**, isolated neuromast associated to a partially perforated scale of the check region. **B**, opercular line and its association to certain perforated scales covering the dorsolateral region of the head. Abb.: **nrm**, neuromast; **op.nl**, opercular line.

The following neuromast lines (or pitlines; Fig 2) are present at least in *Orestias* and the new genus described herein. The ethmoid-supraorbital neuromast line includes many small neuromasts placed one behind the other and adopting the so-called lyre-like pattern in *Orestias* [3,4]. The middle neuromast line or middle pitline is positioned near the posterior margin of the skull roof. It is formed by a few neuromasts. The rostral neuromast line is located slightly below the posterior nostril. It is formed by a few neuromasts. The infraorbital neuromast line comprises a series of neuromasts around the orbit adopting a curved shape anteriorly. This line may be continuous or irregularly discontinuous in certain species. It is not connected with the ethmoid-supraorbital line or the rostral line. The opercular neuromast line is transversely placed in the skin covering the dorsal region of the opercle or slightly above it. This line is irregularly associated to pitted scales in the new genus and species (see Fig 3B). This line is independent and not connected to the preopercular-mandibular line. The preopercular-mandibular line is formed for numerous neuromasts following the preopercle and lower jaw. This line may be connected anteriorly to that of the other side of the head or be disconnected. In addition to the neuromasts forming lines, neuromast fields formed by a few neuromasts can be found in different regions of the head. These can be diagnostic for certain species. Since the presence or absence of neuromast lines can be sexually dimorphic (as in the taxon described here), it is recommended that both sexes be studied. Members of the Orestiini may have short vertical and horizontal neuromast lines on the trunk, which are frequently associated to pitted scales, like the condition observed for the opercular line (see Fig 3B).

### Nomenclatural acts

The electronic edition of this article conforms to the requirements of the amended International Code of Zoological Nomenclature, and hence the new names contained herein are available under that Code from the electronic edition of this article. This published work and the nomenclatural acts it contains have been registered in ZooBank, the online registration system for the ICZN. The ZooBank LSIDs (Life Science Identifiers) can be resolved and the associated information viewed through any standard web browser by appending the LSID to the prefix “http://zoobank.org/”. The LSID for this publication is: urn:lsid:zoobank.org:pub:CD7F1CFB-88D6-4AD4-B153-34B424E79C4F. The electronic edition of this work was published in a journal with an ISSN, and has been archived and is available from the following digital repositories: PubMed Central, LOCKSS.

## Results

### Systematic ichthyology

Teleostei Müller, 1845 (*sensu* Arratia, 1999) [52,53]

Clupeocephala (*sensu* Arratia 2010) [54]

Cyprinodontiformes Berg, 1940 [55]

Cyprinodontidae Wagner, 1828 [56]

Cyprinodontinae Wagner, 1828 [56]

#### Orestiini, new usage

The Tribe Orestiini is diagnosed by the presence of the following characters: Reduced or absent cephalic sensory canals; cephalic sensory system represented by small neuromasts set close together into neuromast lines; pterotic moderately or strongly reduced dorsally leaving a space at the posterodorsal corner of skull roof; robust premaxillary blade with its distal portion broadly expanded; dentary with an expanded ventromedial region and bearing a notch at its medial margin; cartilaginous interhyal; third and fourth pharyngobranchial tooth plates fused to each other; second most anterior branchiostegal ray placed posterior to a deep constriction present in the anterior ceratohyal; thirty to 38 vertebrae; almost horizontally oriented narrow, elongate cleithrum with its largest expansion dorsally; and caudal hypural plate, epural and parhypural symmetrically arranged, Additionally, several loses characterize Orestiini. For instance: vomer absent; dermosphenotic reduced in size or absent; anguloarticular with postarticular process reduced or absent parallel to the retroarticular; teeth on ceratobranchial 4 absent; ventral process of posttemporal absent; first or dorsalmost postcleithrum absent; and pelvic girdles and pelvic fins absent.

**Content and geographical distribution**. Two genera, *Orestias* Valenciennes, 1839 [57] and *Pseudorestias* gen. nov. The two genera inhabit the High Plateau or Altiplano of Peru, Bolivia, and Chile, South America.

#### *Pseudorestias*, gen. nov.

**LSID of the new genus** urn:lsid:zoobank.org:act:82834BD7-505F-4E97-BE8F-D38DDCC8F206

**Generic etymology** *Pseudorestias* in reference to the overall similarities with the genus *Orestias*.

**Generic diagnosis** Same as for type and only species (see below).

**Type species** *Pseudorestias lirimensis* from the Charvinto Creek near to the thermal pond Baños San Andrés, about 6 km E of Lirima village, Chancacolla River, Tarapacá Region, northern Chile.

#### *Pseudorestias lirimensis* sp. nov.

Figs 3–19

Scott et al. [32]: *Orestias* aff. *agassii*: p. 46; mention.

Quezada-Romegialli et al. [58]: *Orestias* sp.: p. 2840; mention and description of mitogenome.

**LSID of the new species** urn:lsid:zoobank.org:act:B0F4C89E-D3F9-43AC-BECB-794657E810DC

**Specific etymology** The specific epithet *lirimensis* refers to the name of the small village Lirima, in northern Chile, that is irrigated by the Charvinto Creek.

**Holotype** MNHNCL ICT-7533, female of 58.56 mm SL. Charvinto Creek near to the thermal pond Baños San Andrés, about 6 km E of Lirima village, in an affluent of the Chancacolla River, Tarapacá Region, northern Chile. 19°51’13.08” S; 68°54’27.52” W at 4,000 m a.s.l. Coll. F. Cruz-Jofré; April 8, 2016.

**Paratypes** MNHNCL ICT-7534, female of 35.16 mm SL. Same data as holotype. MNHNCL ICT-7535, male of 33.3 mm SL. Same data as holotype. MHNV 3254, 2 females, 74.78 and 34.26 mm SL. Chancacolla River, about 7.3 km E of Lirima village, Tarapacá Region, northern Chile. 19°51'30.9'' S, 68°55'7.67'' W. Coll. C. Quezada-Romegialli; October 21, 2016. MHNV 3255, 3 females, 27.1–36.0 mm SL. Charvinto Creek near to the thermal pond Baños San Andrés, about 6 km E of Lirima village, in an affluent of the Chancacolla River, Tarapacá Region, northern Chile. 19°51'12.1'' S, 68°54'27.6'' W. Coll. C. Quezada-Romegialli; October 21, 2016. KUNHM 41384, 5 cleared and stained specimens; 29 and 30 mm SL (males) and 60, 61.73 and 63.76 mm SL (females). Chancacolla River, about 7 km E of Lirima village, Tarapacá Region. Tamarugal Province, Chile; 19°51’24 S; 68°55’02 W, at about 4,000 m a.s.l. Coll. S. Scott and I. Tobar, November 21, 2009.

**Additional specimens** LBUCH 21112009, 12 specimens of 25.3–66.6 mm SL. Chancacolla River, about 7 km E of Lirima village, Tarapacá Region, northern Chile. 19°51’24 S; 68°55’02 W, at about 4000 m a.s.l. Coll. S. Scott and I. Tobar; November 21, 2009. LBUCH 21112009B (= LIR09 in Quezada-Romegialli et al. [58]). Same data as in LBUCH 21112009.

**Diagnosis** *Pseudorestias lirimensis* differs from species of *Orestias* by the following characters: Dorsal cephalic neuromast pattern or ethmoid-supraorbital line represented by an incomplete lyre arrangement of neuromasts in females. There is an absence of these in males. This is distinct from the typical lyre arrangements of small neuromasts in females and males in *Orestias*. Dorsal region of pterotic strongly reduced to its anterior portion leaving a large unossified space at the dorsoposterior corner of the skull roof versus moderately reduction of the pterotic in *Orestias*. Bony anterior and posterior ceratohyals are closely articulated and with a reduced amount of cartilage versus a large gap filled with cartilage between both ceratohyals and ventral to them. Basibranchials are fused into one elongate bony plate versus independent basibranchials. Second pharyngobranchial aligned with pharyngobranchial tooth plate 3+4 versus second pharyngobranchial displaced over pharyngobranchial tooth plate 3+4. Meckel’s cartilage is represented by an anterior, narrow band that broadly expands posteriorly and is reduced to only its anterior part in adults versus one or both regions present and separated by a gap. Middle and distal dorsal-fin radials are ossified in females and partially or not ossified in males versus middle and distal dorsal radials cartilaginous in *Orestias*. Dorsal and anal fins lack simple procurrent rays. All fin rays segmented and only middle rays segmented and with one branching distally. Small spinules are articulated with lateral surfaces of rays for all fins rather than only in dorsal and anal fins. Small rounded or oval scales are distributed in pairs rather than possessing a prominent dorsal ridge of scales between the posterior margin of the head and the insertion of dorsal fin.

#### Description

**Fig 4:**
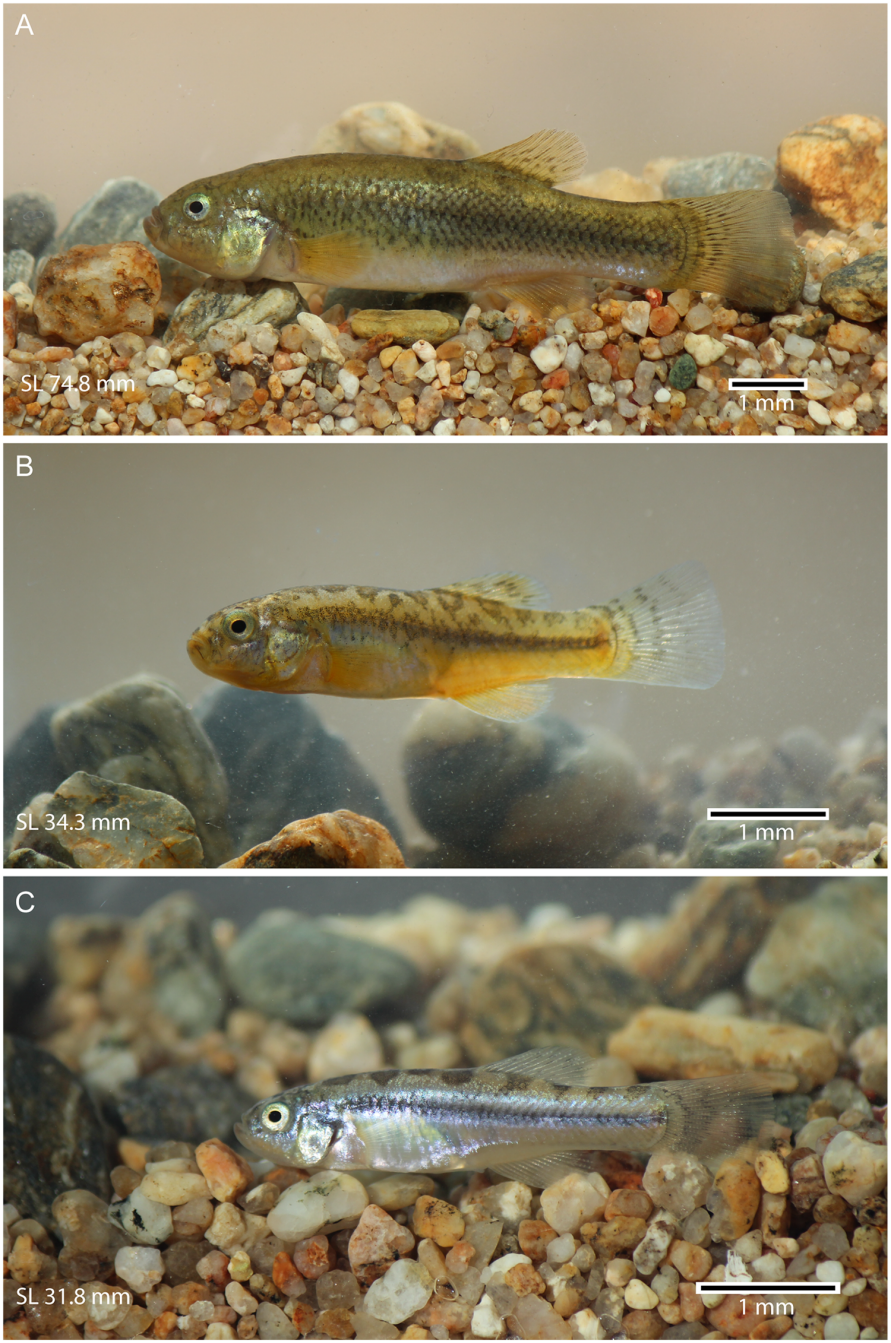
*Pseudorestias lirimensis* gen. et sp. nov. in lateral view. **A**, **B**, and **C** illustrate three females of 74.8 (MHNV 3254), 34.3 (MHNV 3254), and 31.8 mm SL (MHNV 3255), respectively.

**General description** The females are elegantly elongated, with a smooth profile between the orbital rim and dorsal fin (Fig 4A–4C) and become slender posterior to the dorsal fin. The males are slimmer than the females. The overall size of the individuals is small, with the length ranging between 21.9 mm to 74.8 mm SL. The maximum total length of females is approximately 85 mm. This is more than the double of that of males. The mouth is slightly upturned, with an irregular row of pointed unicuspid teeth. The head is moderately large, almost as long as deep. Its length and depth are 26–30% and 21–26% of SL, respectively. The eyes are large, protruding laterally and reaching the primary dorsal profile of head. Their diameter is 24–30% of head length. The greatest body depth is at the dorsal-fin origin (ca. 18–34% SL). It is larger in females, ca 24–34% SL. The insertion of the dorsal fin is placed anteriorly and/or at the same level of the anal fin insertion. Thus, the dorsal and anal fins are placed posterior to the half of body length. The predorsal and preanal lengths are 55–67% and 50–65%, respectively. The caudal peduncle is moderately long and deep (17.6–27.2% and 9.5–16.2% respectively).

The description below explains the diagnostic characters and certain characters which have not been previously described for or are incompletely known in members of the tribe Orestiini from the Altiplano.

**Fig 5:**
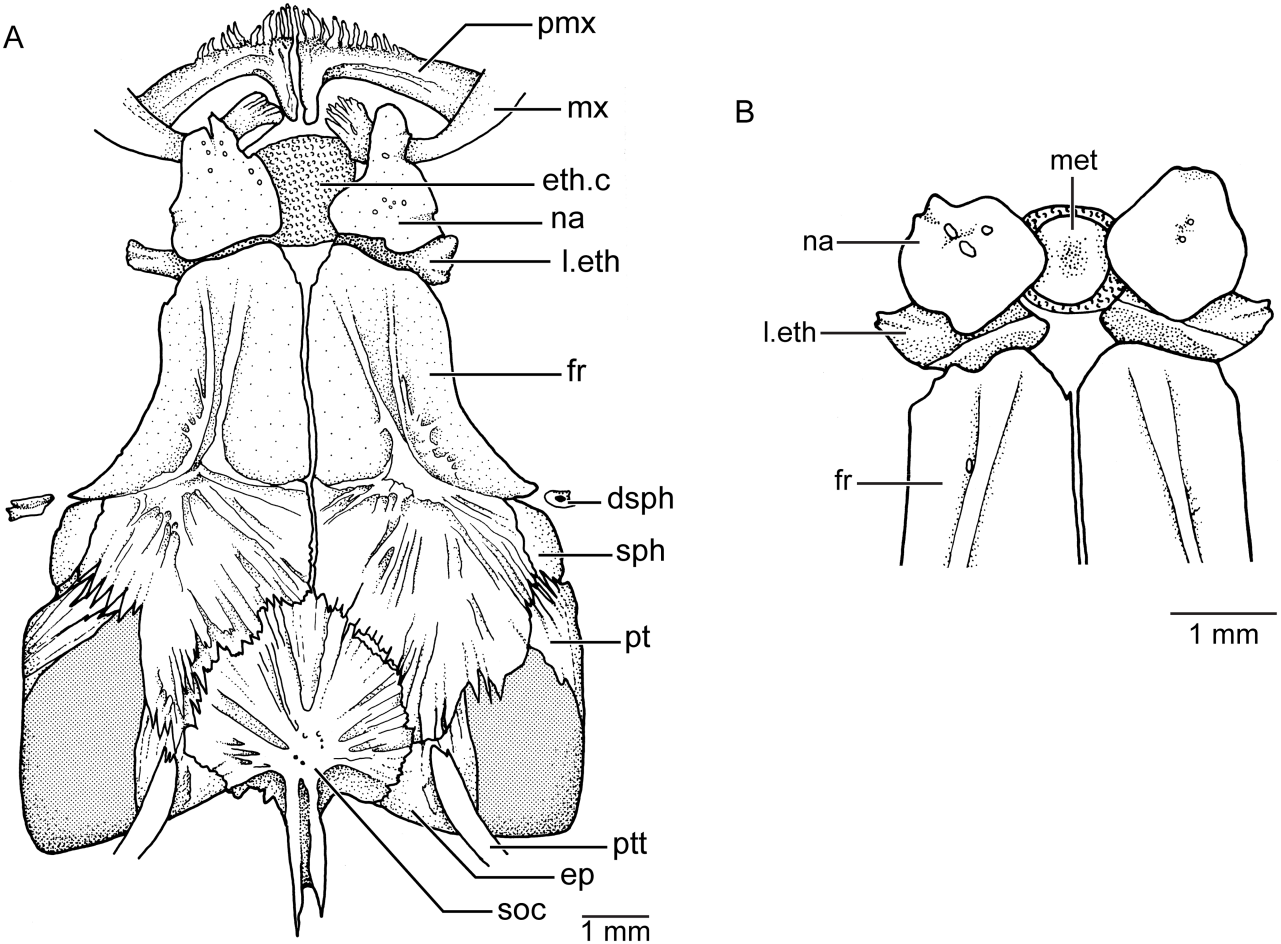
Skull roof bones in dorsal view and their relationships to jaw bones and dermosphenotic in *Pseudorestias lirimensis*. **A**. KUNHM 41384, female of 63.8 mm SL. **B**, KUNHM 41384, male of 30 mm SL. Abbr.: **dsph**, dermosphenotic; **ep**, epiotic; **eth.c**, ethmoid or rostral cartilage; **fr**, frontal bone; **l.eth**, lateral ethmoid; **met**, mesethmoid; **mx**, maxilla; **na**, nasal bone; **pmx**, premaxilla; **pt**, pterotic; **ptt**, posttemporal; **soc**, supraoccipital; **sph**, sphenotic.

**Skull roof** The skull roof (Fig 5A) is characterized by the presence of numerous fine crests extending on the surface of the bones in females, whereas the surface is almost smooth in males. Additionally, the bones are sutured between them by numerous interdigitations of different lengths in females, which are less evident in males. Both sides of the skull roof are asymmetric in all studied specimens.

The posterior part of the skull roof is particularly interesting because of the absence of the parietal bone and the reduction of the pterotic. A well-developed supraoccipital, with a posteriorly directed crest is present in the median region of the skull roof. The crest is not projected above the dorsal cranial level. The supraoccipital sutures laterally with the epiotics that are relatively small and anteriorly with the large frontal bones. Each epiotic has a small elongate crest near its medial margin where the long dorsal arm of the posttemporal articulates. An interesting feature is the reduction of the pterotics at the posterolateral region of the skull roof, which leaves large unossified spaces at the posterodorsal corners of the skull roof. Thus, the dorsal region of the pterotic bone in *Pseudorestias* is restricted to a narrow anterodorsal portion, which sutures with the sphenotic lateroanteriorly and the frontal medially. The large unossified space at the posterodorsal corner of the skull roof in *Pseudorestias* is not a feature related to growth, but a feature characterizing adult females and males. In contrast, species of *Orestias* have a larger development of the pterotic, epiotic, and frontal bones, so that the space left between these bones is considerably smaller (GA pers. obser.).

A small portion of the sphenotic forms the anterolateral corner of the postorbital region of the skull roof. The sphenotic sutures with the pterotic posteriorly and the frontal medially and anteriorly. The largest bone of the skull roof is the frontal bone that is slightly narrower anteriorly than posteriorly where it ends near the posterior margin of the skull roof. Laterally, the bone produces a slightly concave orbital margin that partially protects the protuberant, large eye. Each frontal has a pair of delicate crests anteriorly that seems to follow the course of an absent supraorbital canal. Each frontal bone joins its counterpart throughout a smooth suture (sutura harmonica), whereas the suture with the sphenotic is slightly undulated and the sutures with the pterotic, epiotic, and supraoccipital are dentate and/or serrated.

The nasal bone is placed anteriorly to the anterior margin of the frontal and partially covers the lateral ethmoid. Both nasal bones are broadly separated from each other. Just in between and below them, a large and squared-shaped ethmoid or rostral cartilage is positioned. In addition, this cartilage is also placed between the anterodorsal articular process of the maxillae and the anterior triangular process of the premaxillae.

**Fig 6:**
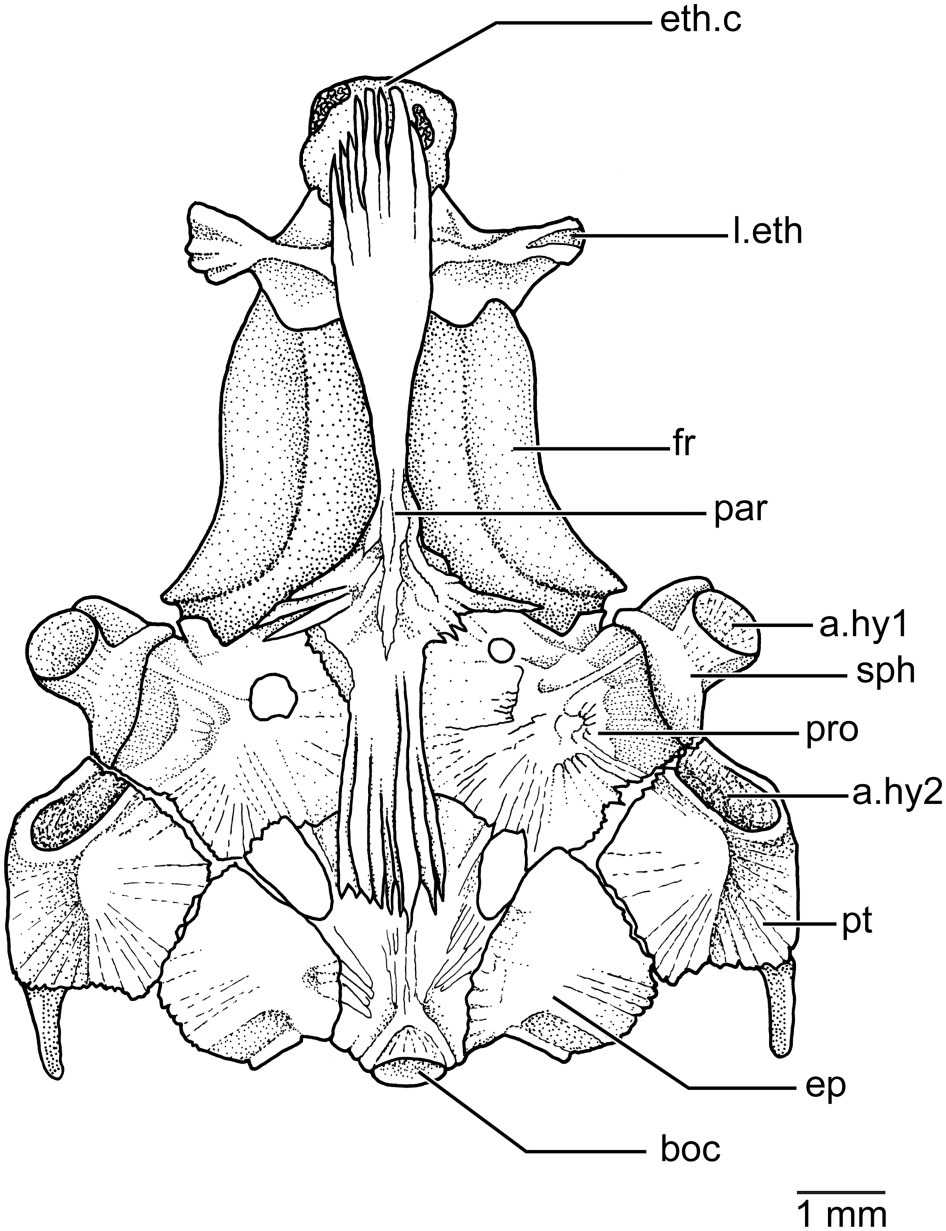
Braincase in ventral view in *Pseudorestias lirimensis* (KUNHM 41384, female of 63.8 mm SL). Abbr.: **a.hy1**, fossa for articulation of the anterior condyle of hyomandibula; **a.hy2**, fossa for articulation of the posterior condyle of the hyomandibula; **boc**, basioccipital; **ep**, epiotic; **eth.c**, ethmoid or rostral cartilage; **fr**, frontal bone; **l.eth**, lateral ethmoid; **par**, parasphenoid; **pro**, prootic; **pt**, pterotic; **sph**, sphenotic.

**Braincase** The ventral side of the braincase (Fig 6) is formed by the median basioccipital, that is a rather short and slightly laterally expanded, bearing posteriorly the articulatory region for the first vertebral centrum. The basioccipital sutures laterally with the epiotics and anteriorly with the parasphenoid and prootics. Two spaces or foramina are left among the basioccipital, prootics, and epiotics. The ventrolateral part of the pterotic forms the posteroventral corner of the braincase and the bone ends in a process or spine directed posteriorly. Ventrally, each pterotic has an elongate articular fossa that articulates with the posterior condyle of the hyomandibular bone. Each prootic sutures with the basioccipital and epiotic posteriorly, the pterotic and sphenotic laterally, and the parasphenoid medially. The surface of the prootic is irregular and it can be perforated or not. It is doubtful if these foramina are for nerves or blood vessels or simply incompletely ossified regions. Each sphenotic has a well-developed ventrolateral region that carries the articular fossa for the anterior condyle of the hyomandibular bone.

The median, elongate parasphenoid expands anteriorly and posteriorly and becomes narrower close to its ascendant processes. The anterior and posterior margins of the parasphenoid are deeply serrated. Irregular serrations are also present between the ascendant process and the medial margin of the prootic. Anteriorly, the parasphenoid sutures with the lateral ethmoids which are slightly expanded medially, ending laterally in irregularly-shaped processes. A vomer is absent in front of the parasphenoid so that the ethmoid cartilage is placed contiguous to the parasphenoid and lateral ethmoids.

In the largest females, the rostral cartilage can develop some small regions of chondroid bone (see Fig 6), whereas a large bone (Fig 5B) ossifies in the ethmoid or rostral cartilage in males. According to our knowledge, such a difference between females and males has not been reported in species of *Orestias* or any other South American cyprinodontiforms.

**Fig 7:**
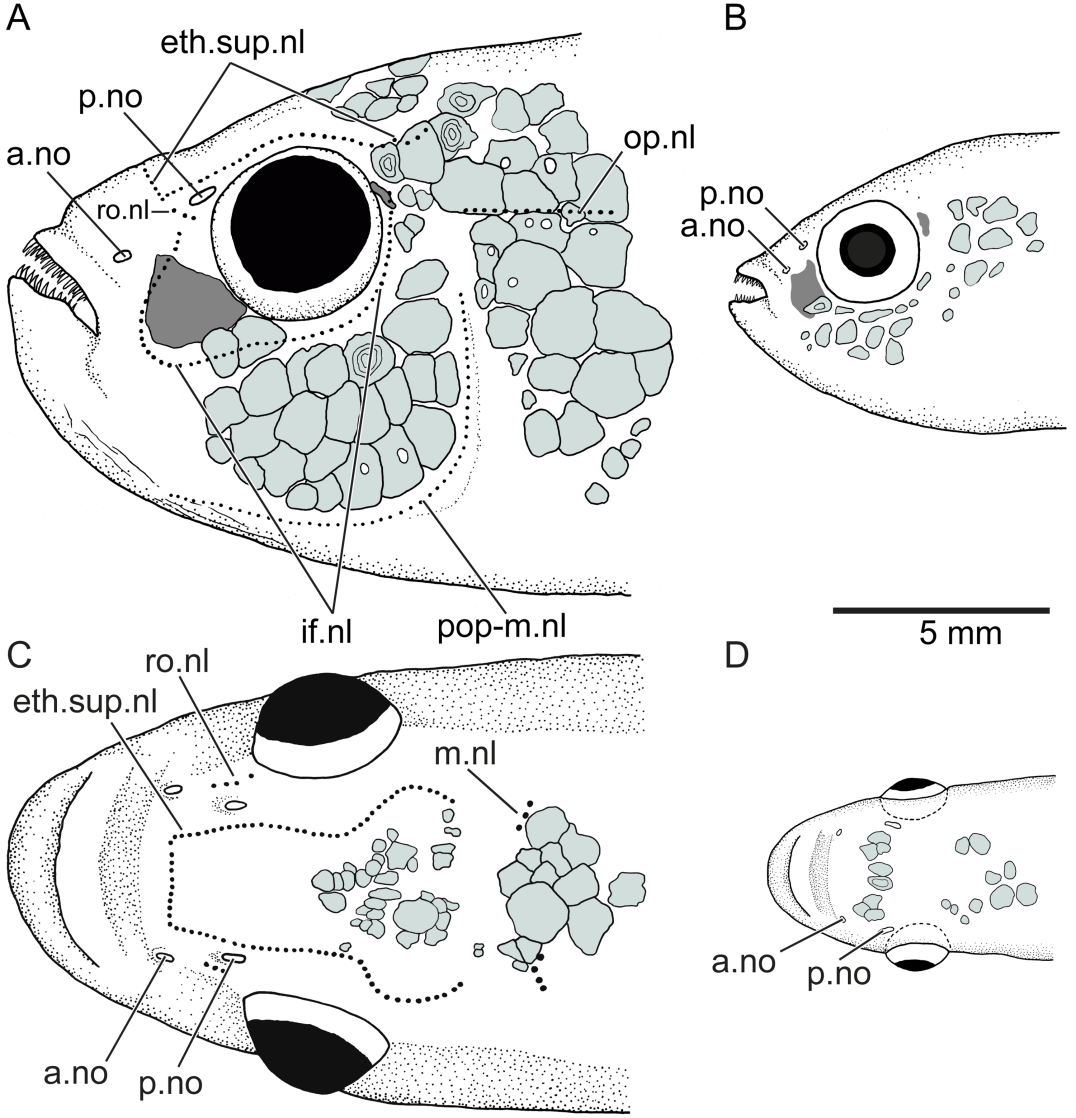
Cephalic neuromast lines and squamation patterns (colored areas) of *Pseudorestias lirimensis* gen. et sp. nov. **A**, head in lateral view (female, 61.7 mm SL; KUNHM 41384). **B**, head in lateral view (male, 30 mm SL; KUNHM 41384). **C**, head in dorsal view (female, 61.7 mm SL). **D**, head in dorsal view (male, 30 mm SL). Abb.: **a.no**, anterior nostril; **eth.sup.nl**, ethmoid-supraorbital neuromast line; **if.nl**, infraorbital neuromast line; **m.nl**, middle neuromast line; **op.nl**, opercular line; **p.no**, posterior nostril; **pop-m.nl**, preopercular-mandibular line; **ro.nl**, rostral neuromast line.

**Cephalic sensory canals and neuromast lines** The cephalic sensory canals are absent. Although a remnant of a groove for the anterior section of the infraorbital canal in the first infraorbital bone (or lachrymal), that is largely expanded and square-shaped, is present, no canal and no sensory pores open in the skin surface. A small dermosphenotic, incomplete ossicle-like (occasionally present in one or both sides of the head; see Figs 5A and 7A), may have a groove, but the posterior section of the infraorbital canal is missing, and no sensory pores open into the skin surface. A dorsoposterior ossicle-like infraorbital is occasionally present in males (see below). The supraorbital, preopercular, and mandibular canals and their pores are absent.

**Fig 8:**
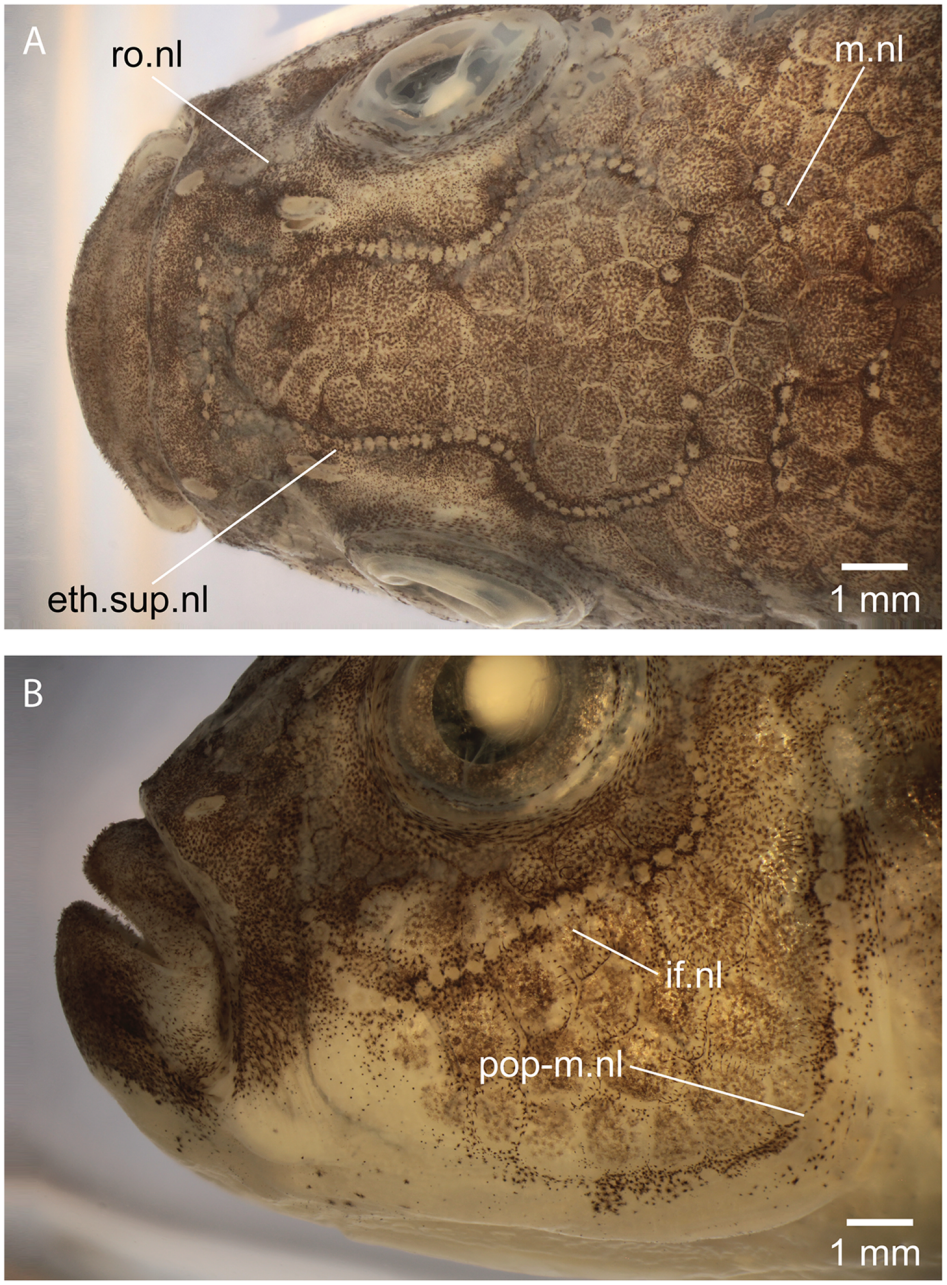
Cephalic neuromast lines of *Pseudorestias lirimensis* gen. et sp. nov. **A**, incomplete head in dorsal view (female, 74.8 mm SL; MHNV 3254) illustrating the ethmoid-supraorbital and middle neuromast lines. **B**, same specimen in lateral view illustrating the infraorbital and preopercular-mandibular lines. Abb.: **eth.sup.nl**, ethmoid-supraorbital neuromast line; **if.nl**, infraorbital neuromast line; **m.nl**, middle neuromast line; **pop-m.nl**, preopercular-mandibular line; **ro.nl**, rostral neuromast line.

The ethmoid-supraorbital line of neuromasts follows the path of the supraorbital canal in other teleosts. Right and left lines join anteriorly producing an incomplete lyre-like pattern (Figs 7A, 7C and 8A) in females. They are absent in males. The small neuromasts are placed one next to the other forming a continuous series that extend up to the level of the sphenotic or just posteriorly. The rostral line is formed by two or three neuromasts commonly, positioned just anteroventral to the posterior nostril. The rostral line does not join the ethmoid-supraorbital and infraorbital lines. The infraorbital line may form a continuous series of neuromast around the orbit (see Figs 7A and 8B) or the line may be shortly interrupted in one or more places. The posterodorsal section of the infraorbital line is not connected with the opercular and preopercular-mandibular lines. The opercular line is horizontally positioned at the level of the dorsal margin of the opercle and its length and number of neuromasts varies among females. Thus, the neuromast lines are discontinuous between them, except for the preopercular-mandibular line. Left and right lines of the latter do not join each other ventromedially. These descriptions correspond to neuromast lines observed in females because neuromast lines were not observed in males (Fig 7B and 7D). Additionally, a few isolated field of neuromasts are irregularly present on the head and also on the body. Short body neuromast lines are placed at almost 90 degrees to the body axis, usually in association with scales with perforated surfaces, similarly to the opercular line illustrated in Fig 3B.

**Fig 9:**
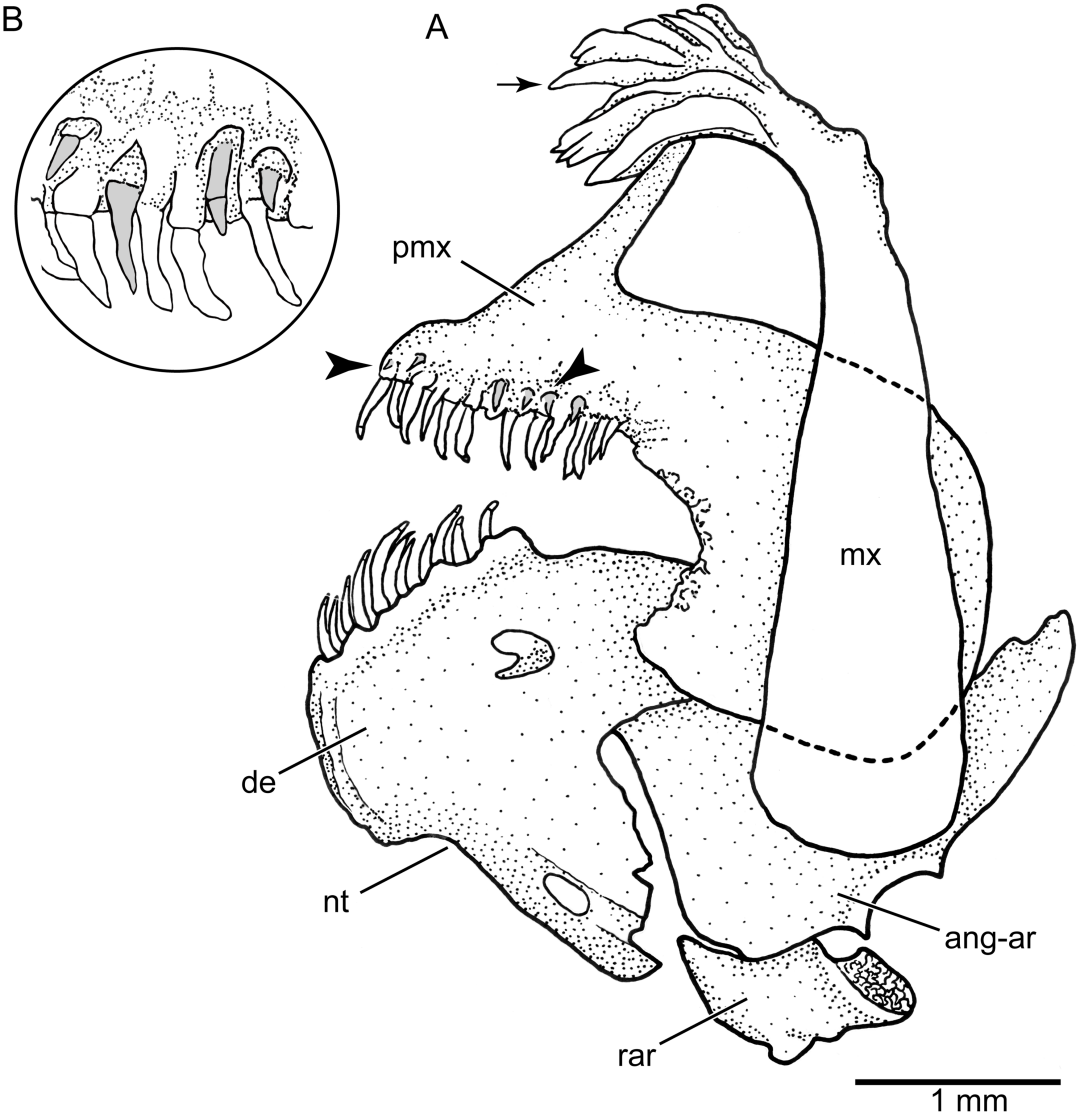
Upper and lower jaws of *Pseudorestias lirimensis* gen. et sp. nov. in lateral view. **A**, upper and lower jaw (KUNHM; female of 63.8 mm SL) and **B**, enlargement of a section of the premaxilla illustrating the position of replacement teeth (see circle insert). Short, small arrow points to the maxillary articular process. Longer and thicker arrowheads point to the region showing replacement teeth. Abb.: **ang-ar**, anguloarticular; **de**, dentary; **mx**, maxilla; **nt**, notch; **pmx**, premaxilla; **rar**, retroarticular.

**Jaws** The upper jaw (Fig 9A) is slightly upturned. The premaxilla has an elongate, narrow and sharp ascendant process that may be distally covered by the maxillary anterodorsal process. The robust premaxillary blade is markedly curved, posteroventrally expanded (Fig 9A), and covered by the maxilla like in *Orestias* (see fig 20 in [3],[4]; GA pers. obser.). Females of ca. 60 mm SL have one row of 12 to 19 unicuspid conic teeth. Males of ca. 30 mm SL have one row of ca. 9 conic teeth. The external surface of the premaxilla (near the oral border) is irregularly thickened, leaving fossae and crests of different sizes that house replacement dentition irregularly placed (Fig 9B), which may be considered an incomplete or irregular outer row. At least six replacement teeth of different sizes are observed in the female illustrated in Fig 9A. The first replacement tooth is just at the symphyseal margin. It is a tiny conic tooth that is easily overlooked and is represented mainly by the acrodin cup. It is just anterodorsal to the first tooth that seems to be the longest and curved of the tooth series. The teeth are irregular in their length and in their curvature.

The maxilla is markedly curved near its articulatory region forming an angle of about 90 degrees with the maxillary blade (Fig 9A). The maxillary blade is placed almost parallel to the anterior border of the head which is largely built by the dentary. The maxillary blade is elongated and becomes gently expanded and curved ventrally, a feature also shared with some species of *Orestias* (see fig 20A–B in [3]; GA pers. obser.). [A lobe-shaped and short ventral process of the maxilla was proposed as a synapomorphy of *Orestias* (= Orestiini) by [4]). However, a long, narrow process of the maxilla is a feature present in numerous species of *Orestias* (see [3]; GA pers. obser.).] The maxillary articulatory region has a large, broadly expanded anterodorsal process with its anteromedial margin irregularly serrated in large females and with thin bony flanges extending between the serrations in males and some females. Left and right processes do not meet with each other at midline (Fig 5A). A heavily ossified, styliform ventral process is present in both females and males. An elongate, flat and broad nasal bone extends on the process and partially covers it (Fig 5A).

**Fig 10:**
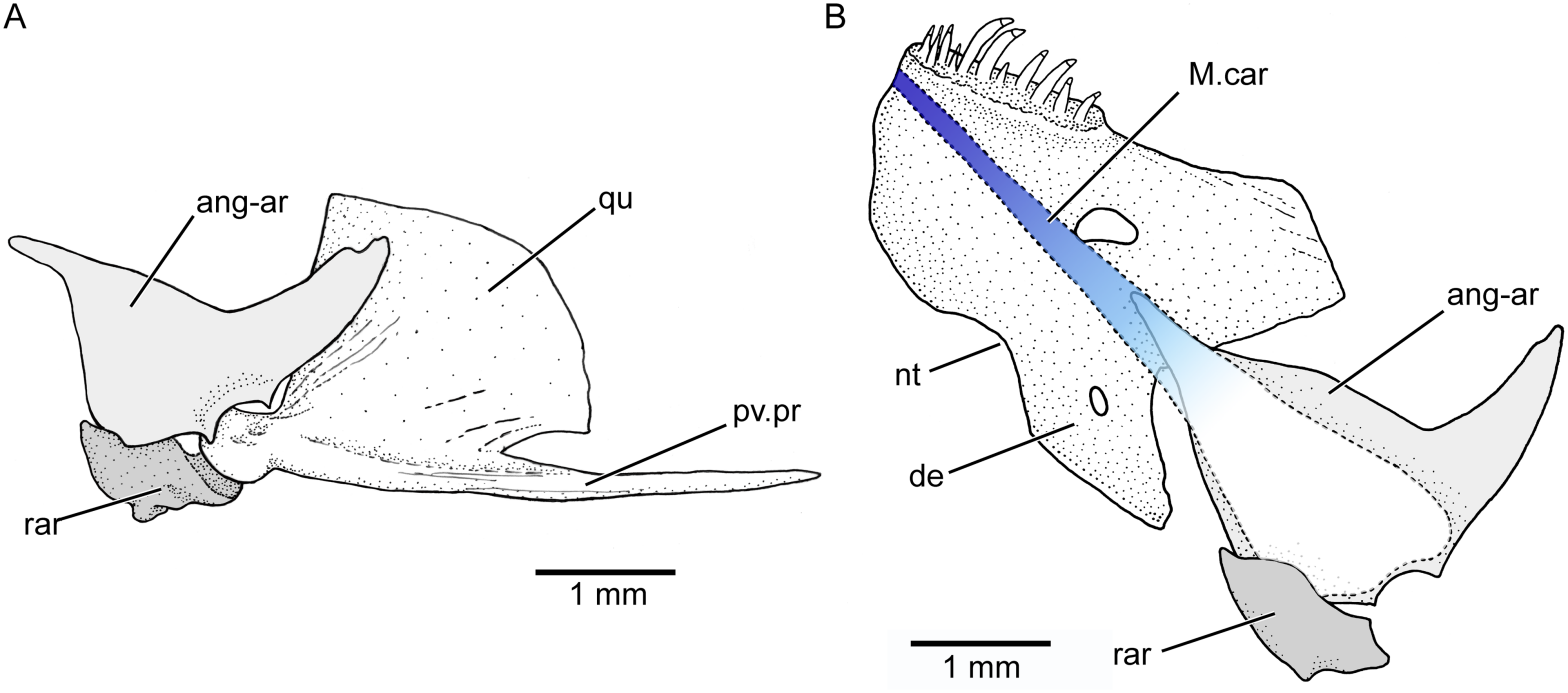
Articulation of the lower jaw with quadrate and position of Meckel’s cartilage. Females of 61.73 mm SL and 63.8 mm SL (KUNHM 41384). **A**, posterior part of jaw in lateral view and its articulation with quadrate. Note the contribution of the retroarticular as part of the articular fossa. **B**, jaw in medial view illustrating the Meckel cartilage. Abb.: **ang-ar**, anguloarticular; **de**, dentary; **M.car**, Meckel’s cartilage; **nt**, notch; **qu**, quadrate; **pv.pr**, posteroventral process; **rar**, retroarticular.

The dentary (Figs 9A and 10B) is a short and massive bone, with an enlarged ventromedial region characteristic of members of the tribe Orestiini [2,4,herein]. This medial region has a deep notch at its medial margin (Figs 9A and 10B) that is also present in *Orestias* ([3,4]; GA pers. obser.). The dentary carries one row of unicuspid conic teeth close to the symphysis. Females of about 60 mm SL carry eleven or 12 teeth. A few replacement teeth are positioned just medially contrary to the situation present by the premaxillary replacement teeth. Males of about 30 mm SL have one row of ca. six to nine conic teeth. The coronoid process of the jaw is mainly formed by the posterodorsal region of the dentary, which ends in a blunt tip, a condition also present in some species of O*restias* (e.g., fig 20A in [3]; GA pers. obser.). In contrast, an elongated posterodorsal process of the dentary was proposed as a character of *Orestias* (= his Orestiini) by Costa [4], a feature that is only present in some species. The coronoid process is intraspecifically variable, being very tall in males. A weak joint exists between the dentary and the anguloarticular. The latter lacks a well-developed posterior process (postarticular process) so that the small retroarticular bone (Fig 10A) is part of the articulation with the quadrate—an unusual condition in clupeocephalan teleosts. The retroarticular bone is not vertically expanded, a character proposed for *Orestias* [4]. The lower jaw is short and its articulation with the quadrate is anteroventral to the anterior margin of the orbit. The shortening of the jaw is accompanied by an elongation of the posteroventral process of the quadrate (Fig 10A) as well as the symplectic to support the lower jaw.

The Meckel’s cartilage (Fig 10) is continuous along the medial side of each lower jaw. It keeps the early ontogenetic condition to be united with its counterpart at the symphyseal region even in the largest females here studied. [This represents a completely different condition to other cyprinodontiforms as illustrated by Parenti [2,3] and Costa [4], with the Meckel’s cartilage not reaching the symphyseal region.] It is a moderately thin band of hyaline cartilage at its anterior end on the dentary and expands posteriorly, extending onto the anguloarticular and retroarticular. The posterior section of the cartilage is almost completely resorbed in large females although its limits still can be observed. This pattern differs from that described or illustrated for some species of *Orestias* (e.g., fig 43B in Parenti [2]; fig 5B in Costa [4]; GA pers. obser.)

**Fig 11:**
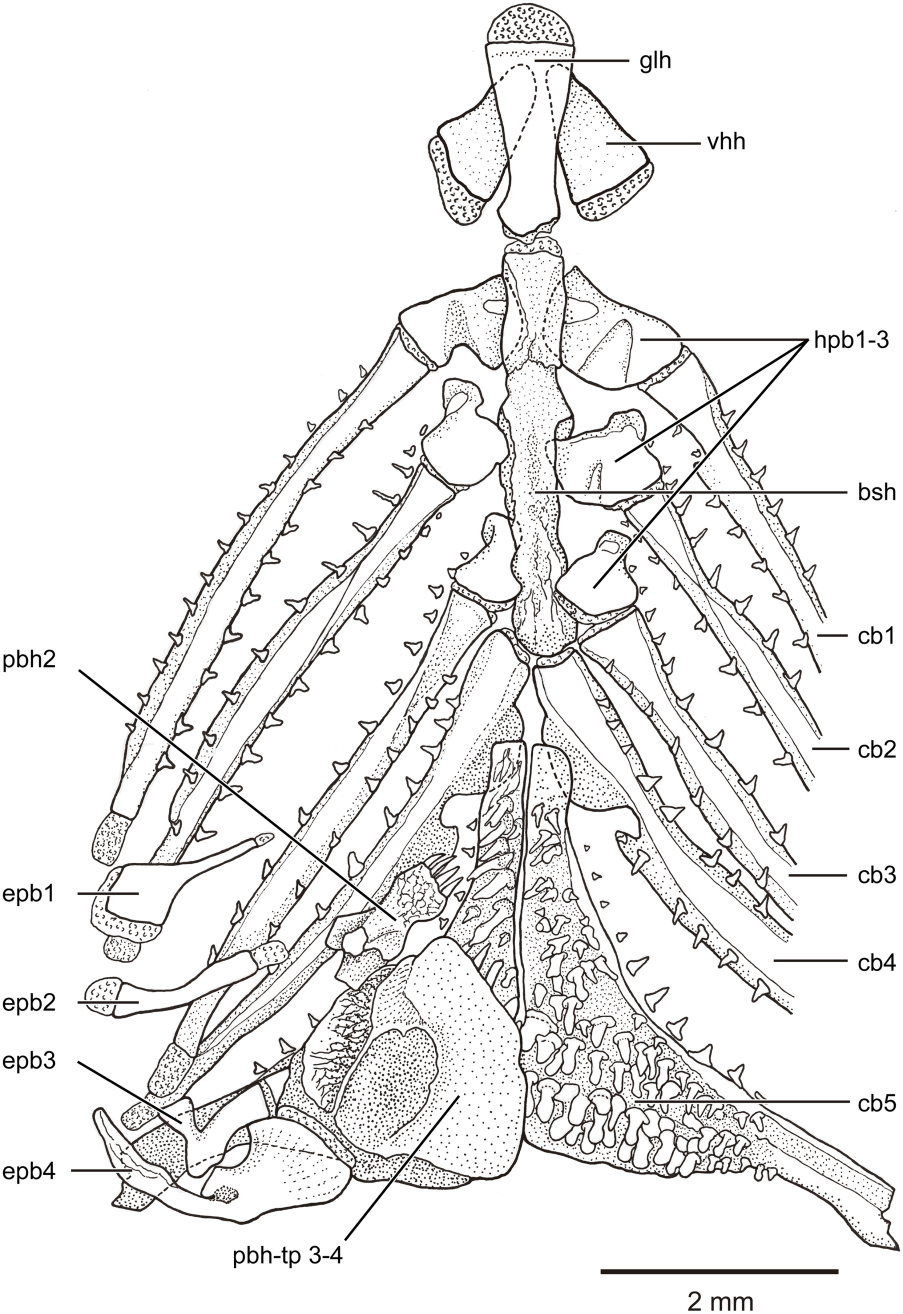
Branchial arches and teeth of *Pseudorestias lirimensis* gen. et sp. nov. Branchial arches in dorsal view (left side) with epibranchials and pharyngobranchials removed on the right side. Female of 61.7 mm SL (KUNHM 41384). Abbr.: **bsh**, basibranchial plate; **cb1-5**, ceratobranchials 1–5; **epb1-4**, epibranchials 1–4; **glh**, glossohyal; **hpb1-3**, hypobranchials 1–3; **pbh2**, pharyngobranchial 2; **pbh-tp 3–4**, tooth plate of pharyngobranchials 3–4; **vhh**, ventral hypohyal.

**Branchial and hyoid arches** The branchial arches (Fig 11) are characterized by the presence of long and narrowly paired ceratobranchials 1–4 and small, slightly triangular paired hypobranchials 1–3, which lack tooth plates. Hypobranchial 4 is missing and ceratobranchial 4 articulates with the basibranchial region by a narrow cartilage. The basibranchial region is represented by a long, narrow and well-ossified bone (interpreted here as result of the fusion of independent basibranchials). The basibranchial plate joins anteriorly with the median glossohyal, laterally with paired hypobranchials 1–3, and posteriorly with paired ceratobranchials 4. Paired ceratobranchials 5 do not join with the basibranchial plate due to the position of the proximal tips of ceratobranchials 4 that displace the ceratobranchials 5 posteriorly. Each ceratobranchial 4 has a ventral bony flange or crest that extends below the anterior tip of ceratobranchial 5. The medial margin of each ceratobranchial 4 presents a well-developed notch. Teeth are absent on ceratobranchial 4. Both ceratobranchials 5 are not fused with each other, but are joined medially by a straight joint. Each ceratobranchial 5 is modified into an almost triangular bone with its dorsal surface covered irregularly by teeth of different sizes and shapes (Fig 11). Although many of the teeth are unicuspid, a few bicuspid teeth can be present. Most anterior teeth are mainly conic with a small acrodin tip, but they become larger and blunt with molariform-like aspect posteriorly (especially in the most posterior teeth). All or most of them present the small triangular-shaped acrodin tip. Each ceratobranchial 5 presents a well-developed flange or crest ventrally, similar to that present on the ventral side of ceratobranchial 4, but larger.

The dorsal part of the branchial arches includes epibranchials and pharyngobranchials. Epibranchials 1–4 have very different shapes and sizes (see Fig 11), being epibranchials tube-like and broadly separated from their counterparts. Epibranchial 1 is a short bone, rhomboidal-shaped distally and narrowly projected proximally. The bar-like epibranchial 2 is short, thin, and slightly curved. The small V-like epibranchial 3 has two arms placed in an angle of 90 degrees to each other. This epibranchial joins ceratobranchial 3 laterally and pharyngobranchial 3 medially throughout elongate cartilages. Epibranchial 4 is larger and longer than the other three elements of the series and joins pharyngobranchial tooth plate 3+4. An ossified pharyngobranchial 1 has not been observed. Pharyngobranchial 2 is irregularly shaped, heavily ossified, and bearing a tooth plate on its ventral surface. Its teeth are unicuspid, conic, and cover completely the ventral surface of the bone. Pharyngobranchial 2 is aligned with pharyngobranchial 3 and pharyngobranchial tooth plate 3+4 (Fig 11). [This condition differs from that cited and/or illustrated for members of *Orestias* [e.g., 3,4] and Cyprinodontinae [3] with pharyngobranchial 2 dorsally displaced over pharyngobranchial tooth plate 3+4.] Pharyngobranchials 3–4 are represented by a massive, heavily ossified toothed plate that is densely covered by ca. 100 conic teeth (in large females) bearing each a tip of acrodin.

Gill rakers (Fig 11) of different sizes are aligned along the anterior and posterior margins of each branchial arch. Twenty-three to 25 gill rakers are associated with the first branchial arch of the left side; twelve to 14 on the anterior margin and ten or eleven on its posterior margin. The right first branchial arch has a similar number of gill rakers or higher.

**Fig 12:**
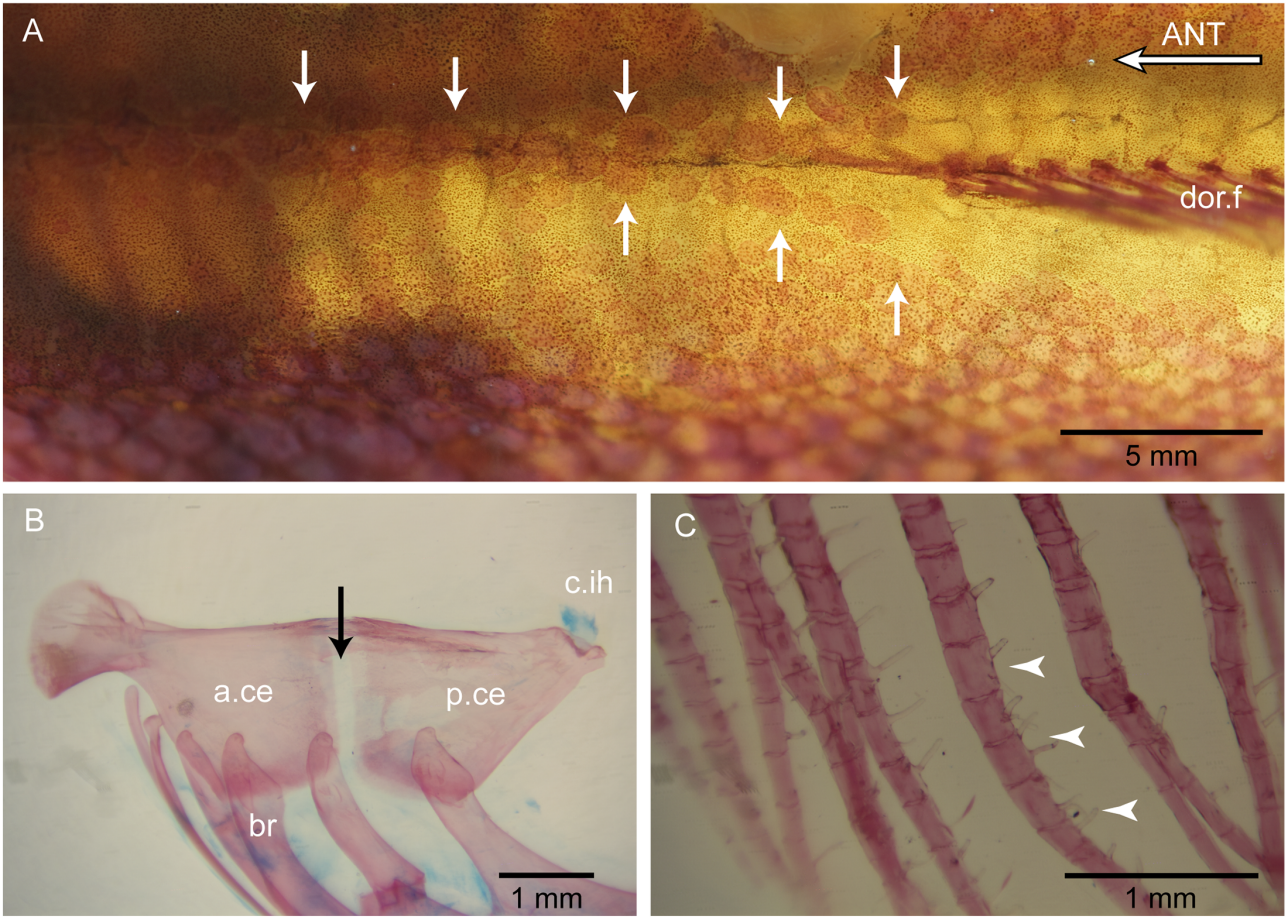
Scales, hyoid arch, and bony spinules in fin rays of *Pseudorestias lirimensis* gen. et sp. nov. (female, 61.7 mm SL; KUNHM 41384). **A**, scales (indicated by white arrows) in the median region between the posterior part of the head and insertion of dorsal fin. **B**, hyoid arch and branchiostegal rays. Black arrow points to the narrow articulatory area between ceratohyals. **C**, spinules (indicated by arrowheads) articulated with the lateral surfaces of the anal fin rays. Abb.: **a.ce**, anterior ceratohyal; **ANT**, anterior; **br**, branchiostegal rays; **c.ih**, cartilaginous interhyal; **dor.f**, dorsal fin; **p.ce**, posterior ceratohyal.

Each hyoid arch (Fig 12B) is composed by a ventral hypohyal, anterior and posterior ceratohyals partially fused dorsally, and a cartilaginous interhyal similar to that present in *Orestias*. The articulatory region between anterior and posterior ceratohyals is narrow. It is filled with a small quantity of cartilage that extends in a small, narrow cartilage ventrally. This certainly is a different condition to the one present in the genus *Orestias* (see [3,4], with a broader articular cartilage that extends largely ventrally to both ceratohyals (compare with fig 6A–B in Parenti [3]).

Six branchiostegal rays are present (Fig 12B). Branchiostegal 1 is attached to the posterior ceratohyal. Branchiostegal 2 is near or in the articulation between posterior and anterior ceratohyals. The other four elements attach with the anterior ceratohyal. The two most posterior branchiostegal rays (1 and 2) are the longest and the largest and are cimitarra-like. Branchiostegals 3 to 6 are elongate and the two most anterior ones are the narrowest and shortest of the series. The second most anterior branchiostegal ray (Fig 12B) is placed just posterior to the deep notch present in the anterior ceratohyal, a character interpreted as a synapomorphy of Orestiini by Costa [4] and herein.

**Fig 13:**
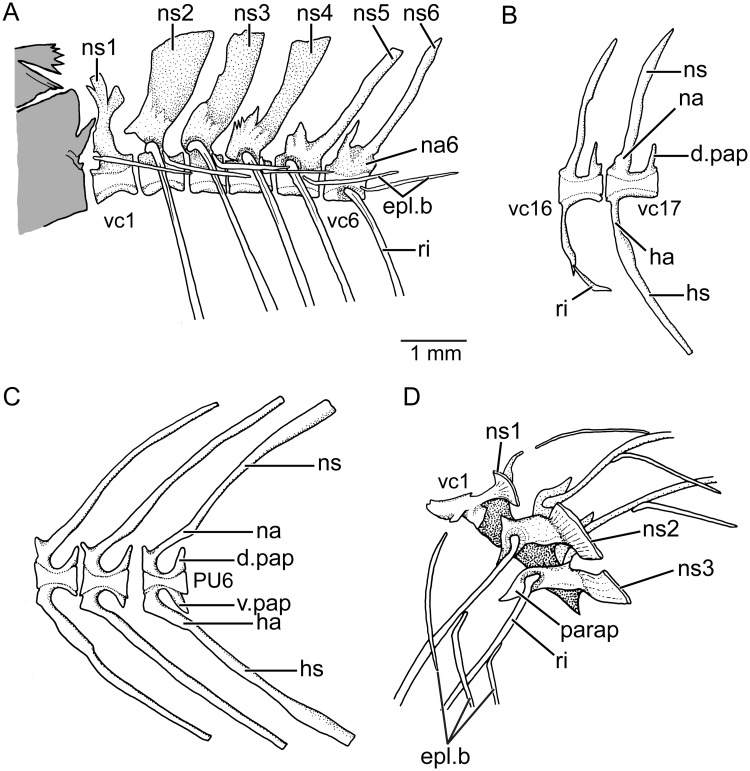
Vertebral column and associated elements (KUNHM 41384; female of 61.7 mm SL). **A**, anteriormost abdominal vertebrae in lateral view (note that the posterior region of braincase is colored in gray). **B**, vertebrae of middle body region. **C**, posterior caudal vertebrae. **D**, abdominal vertebrae 1–3 in a slightly inclined laterodorsal view illustrating the relationships of ribs and epicentrals. Abh.: **d.pap**, dorsal postzigapophysis; **epl.b**, epipleural bones; **ha**, haemal arch; **hs**, haemal spine; **na**, neural arch; **na6**, neural arch of vertebra 6; **ns**, neural spine; **ns1-6**, neural spines of vertebrae 1–6; **parap**, parapophysis; **PU6**, preural centrum 6; **ri**, ribs; **v.pap**, ventral postzigapophysis; **vc1, 6, 16, 17**, vertebral centrum 1, 6, 16, 17.

**Vertebral column and associated elements** There are 34 to 36 vertebrae excluding the terminal caudal centrum, a count that is within the range of Orestiini after Costa [4] and herein. Twenty or 21 are caudal vertebrae. The caudal centra are square-shaped with neural and haemal arches placed in their anterior half (Fig 13C). The arches are fused to the centra except for the haemal arch of preural centrum 1. Remnants of the arch are present on the proximal region of the parhypural. Abdominal centra 2–4 (Fig 13A) bear expanded neural arches and spines. The expansion of the neural spines diminishes starting the fifth vertebra. Among the first three vertebrae, the second centrum bears the broadest spine (Fig 13A and 13D). The abdominal vertebrae bear well-ossified, large, and leaf-like parapophyses fused to the ventrolateral walls of each centrum. They articulate with 15 or 16 pairs of ribs that are markedly curved proximally, being the last one a tiny one (Fig 13B). The first centrum does not bear a rib although the space is partially occupied by the first epipleural bone that is smaller than the following epipleurals. A short ligament connects it to the first centrum. Slender but well-ossified epipleural bones (Fig 13D) are associated to the dorsal region of the ribs and not to the neural arches. Epineurals and epicentrals are absent as well as supraneurals. The caudal vertebrae are characterized by the presence of well-developed and sharp apophyses (associated with the anterior region of the neural arches) and well-developed and sharp posterior apophyses (associated to the dorsoposterior region of the centrum).

**Fig 14:**
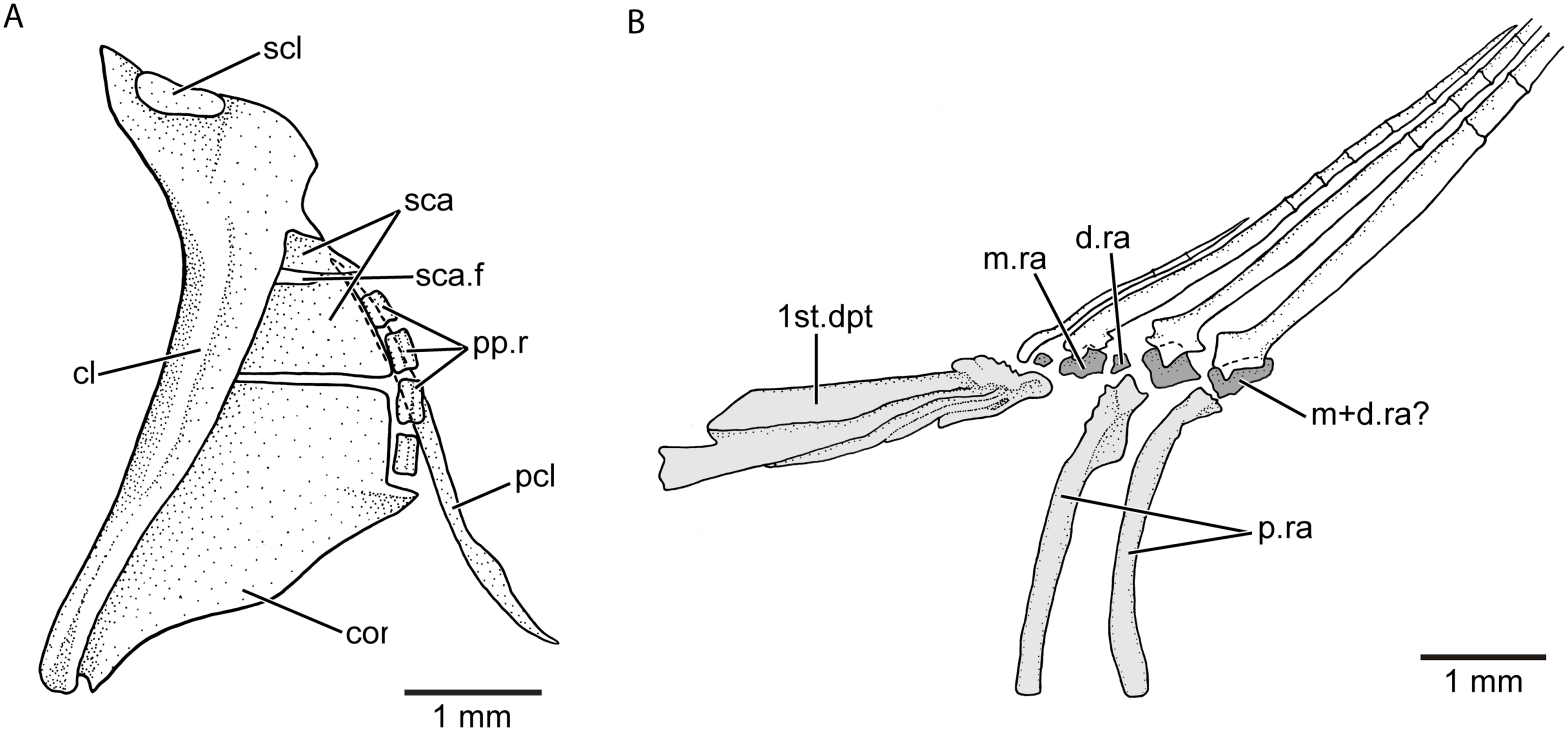
Pectoral girdle with posttemporal excluded (A) and bases of anterior dorsal fin rays and their supporting elements (B) of *Pseudorestias lirimensis* gen. et sp. nov. Abb.: **cl**, cleithrum; **cor**, coracoid; **d.ra**, distal dorsal radial; **m+d.ra?**, middle plus distal radial?; **m.ra,** middle radial; **pcl**, postcleithrum; **pp.r**, proximal pectoral radials; **p.ra**, proximal dorsal radials; **sca**, scapula; **sca.f**, scapular foramen; **scl**, supracleithrum; **1st.dpt**, first dorsal pterygiophore.

**Pectoral fin** The spatula-like or oval-like pectoral fin is ventrolaterally placed below the mid-flank. It has eleven to 14 rays (S2 Table). The middle rays have only one branching that is comparatively deeper than those in the dorsal and anal fins. The marginal rays are thinner and shorter than the middle rays. All rays appear very delicate and fragile. Small spinules are articulated to the rays in variable quantity in males and females (a feature that has not been reported about the pectoral fin of species of *Orestias*). The pectoral girdle (Fig 14A) has a similar morphology to that described and illustrated for species of *Orestias* (see fig 17 in [2])], with the posttemporal represented only by its dorsal arm, a small, scaly-like supracleithrum at the dorsal margin of cleithrum, a cleithrum almost vertically oriented with its broader region dorsally, a large and broad coracoid reaching the ventral tip of the cleithrum, and lacking postcleithrum 1. There is a long, styliform-like postcleithrum (Fig 14A), which is dorsally hidden by the posterior region of the cleithrum, scapula, and proximal radials and is free ventrally (not covered by other elements of the girdle). It is unclear whether the long postcleithrum is only one element (postcleithrum 3) or the result of the ontogenetic fusion of postcleithra 2 and 3. Four small and square-shaped proximal radials are present.

**Unpaired fins** The dorsal and anal fins (Fig 4) are placed slightly posterior to the half of the SL. The base of both fins follows smoothly the profile of the body giving the fish a gently elongate body shape. Both fins have slightly rounded profiles. A narrow area without scales is observed along the base of the dorsal and anal fins. Both fins bear spinules associated to the lateral surfaces of the rays with some of them conspicuously long (Fig 12C).

The dorsal fin has eleven to 16 rays (commonly 14; S2 Table). Males have fewer rays, commonly eleven. The rays are characteristic: the first four or five rays are thin and only segmented. They are followed by four or five rays which are segmented and finely branched distally (only one distal dichotomy in the largest females). The last four rays (including the last pair) are only segmented. Each dorsal ray is articulated with one pterygiophore (Fig 14B), except for the first two and the last two rays that articulate with one pterygiophore.

The first pterygiophore (Fig 14B) is almost parallel to the body axis and is straight. [This is different to the condition found in *Orestias*—with slightly bent first dorsal and anal pterygiophores—after Costa [4].] All other proximal radials are oriented ventrally in *Pseudorestias*. The enlarged first pterygiophore bears the first two dorsal rays. Middle and posterior radials of the first pterygiophore are ossified. The following pterygiophores are associated each with one ray. Each proximal radial articulates with one ossified, elongate element that seems to be the result of the fusion between the middle and distal radials. At the middle region of the fin, there are middle radials that are partially ossified and are followed by cartilaginous distal radials. The last few two or three rays are supported by cartilaginous middle and distal radials. We hypothesize that older specimens may have all middle and distal radials ossified. The ossification of the middle and distal radials appears to be sexually dimorphic, because males, apparently, do not ossify them. [In contrast, cartilaginous dorsal and anal radials are present in *Orestias* [3,4].]

The anal fin has twelve to 15 rays that are similar to the dorsal rays. The first three to five rays are only segmented. They are followed by eight to ten rays that are segmented and finely branched distally (only one dichotomy as with the dorsal rays). The last pair, that is supported by one pterygiophore, is only segmented, being one ray much longer than the other. The ossification of the middle radials is similar to that of the dorsal radials but frequently all distal radials are ossified. This is the condition observed in females. In males, the middle and distal radials are commonly unossified.

**Fig 15:**
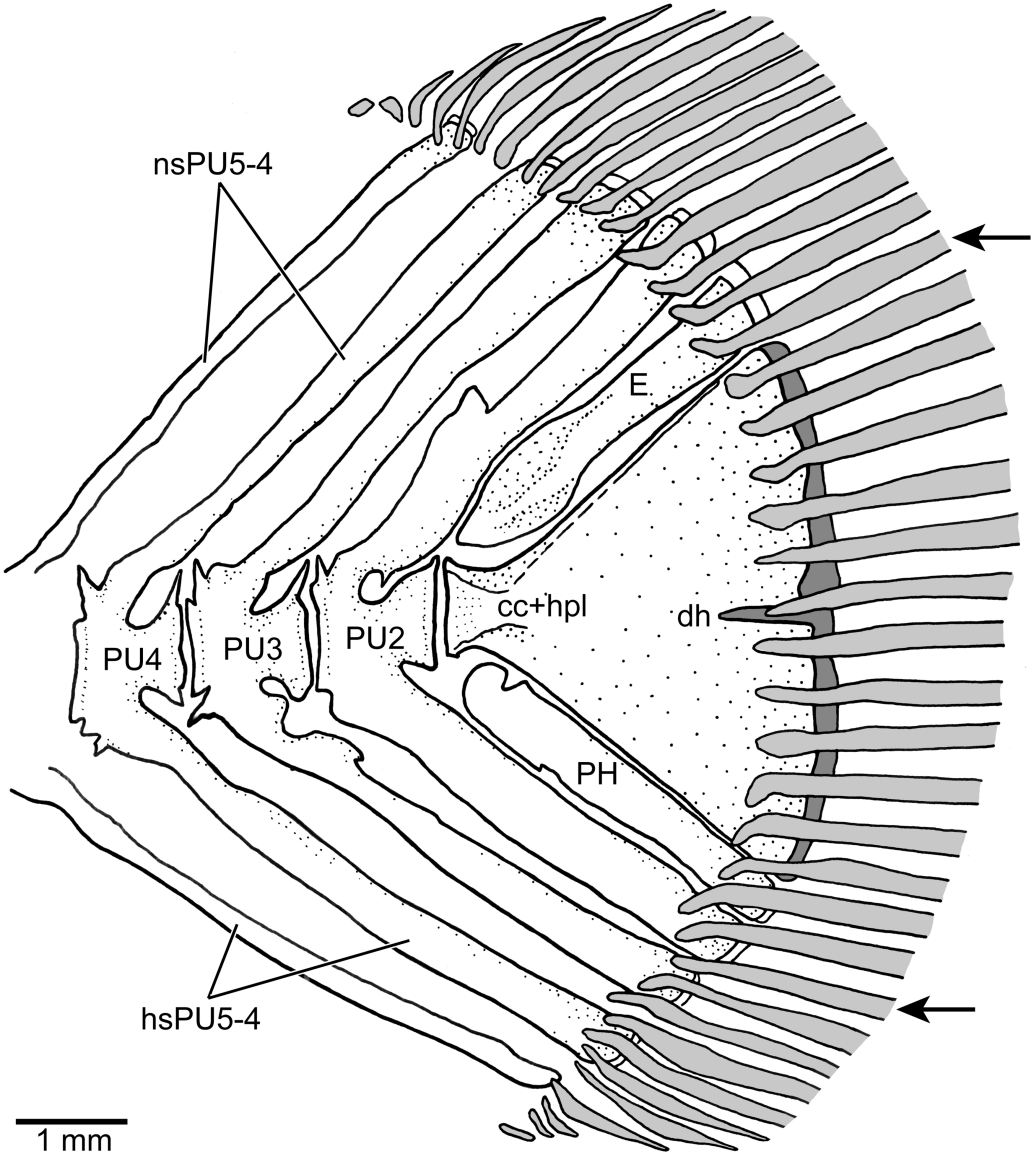
Caudal skeleton in lateral view of *Pseudorestias lirimensis* gen. et sp. nov. Female of 61.7 mm LS (KUNHM 41384). Arrows point to the first and last principal rays. Abb.: **cc+hpl**, compound centrum and fused hypurals; **dh**, reduced hypural diastema; **E**, epural; **hsPU5-4**, haemal spines of preural centra 5, 4; **nsPU5-4**, neural spines of preural centra 5–4; **PH**, parhypural; **PU4-2**, preural centra 4–2.

The caudal fin has an overall rounded profile with its posterior margin straight, or truncate, or slightly rounded. There are 40 or 41 caudal rays including 16 principal rays. The caudal endoskeleton is overly similar to that described and illustrated for *Orestias* (e.g., [3,18,43]), with an almost symmetric placement of rays in relation to the hypural plate centrally placed with epural dorsally and parhypural ventrally (Fig 15). The fin rays are supported by five preural vertebrae that are heavily ossified with their neural and haemal arches fused to the anterior half of the centrum. The posterior half is slightly narrower but produces long, sharp dorsal apophyses. The neural and haemal spines of preural centrum 2 are broader than other spines. There is one epural that is oval-shaped ventrally. It occupies almost the complete space left between the neural spine 2, the dorsal margin of the terminal centrum, and the hypural plate. The proximal part of the parhypural is not connected to the centrum or urostyle. The parhypural is a broad element occupying the space between the haemal spine 2 and the anteroventral margin of the hypural plate. The terminal centrum (including preural 1) is fused in early ontogeny to an unknown number of ural centra and to the hypurals (independent uroneurals are absent in adults). No evidence of sutures is observed in the specimens examined. Without larval stages, we cannot provide information on the ontogenetic development and composition of this plate and of the presence or complete absence of uroneurals. At the mid-region of the posterior margin of the plate a narrow small hypural diastema is present. Interneural and interhaemal cartilaginous plates placed in the spaces between neural spines of preural centra 6–5 and 5–4 and between the haemal spines of preural centra 6–5, 5–4, and 4–3 (which are known in species of *Orestias*), are not present in the largest females of *Pseudorestias lirimensis*. The cartilages that are irregularly present in small individuals get resorbed during growth, due to the broadening of the spines that reduce the space of the cartilaginous plates or to the loss of cartilage.

The caudal fin has 14 or 15 dorsal procurrent, 16 principal, and ten or eleven ventral procurrent rays. The bases of the rays that are associated to the dorsal and ventral leading margins of the fin are bent in opposite directions. Those of the dorsal region are bent dorsally, whereas those of the ventral region are bent ventrally. The bases of the principal rays are variably associated with small spinules as those present in other fins. Small, thin scales extend irregularly on the bases of the principal caudal rays.

**Fig 16:**
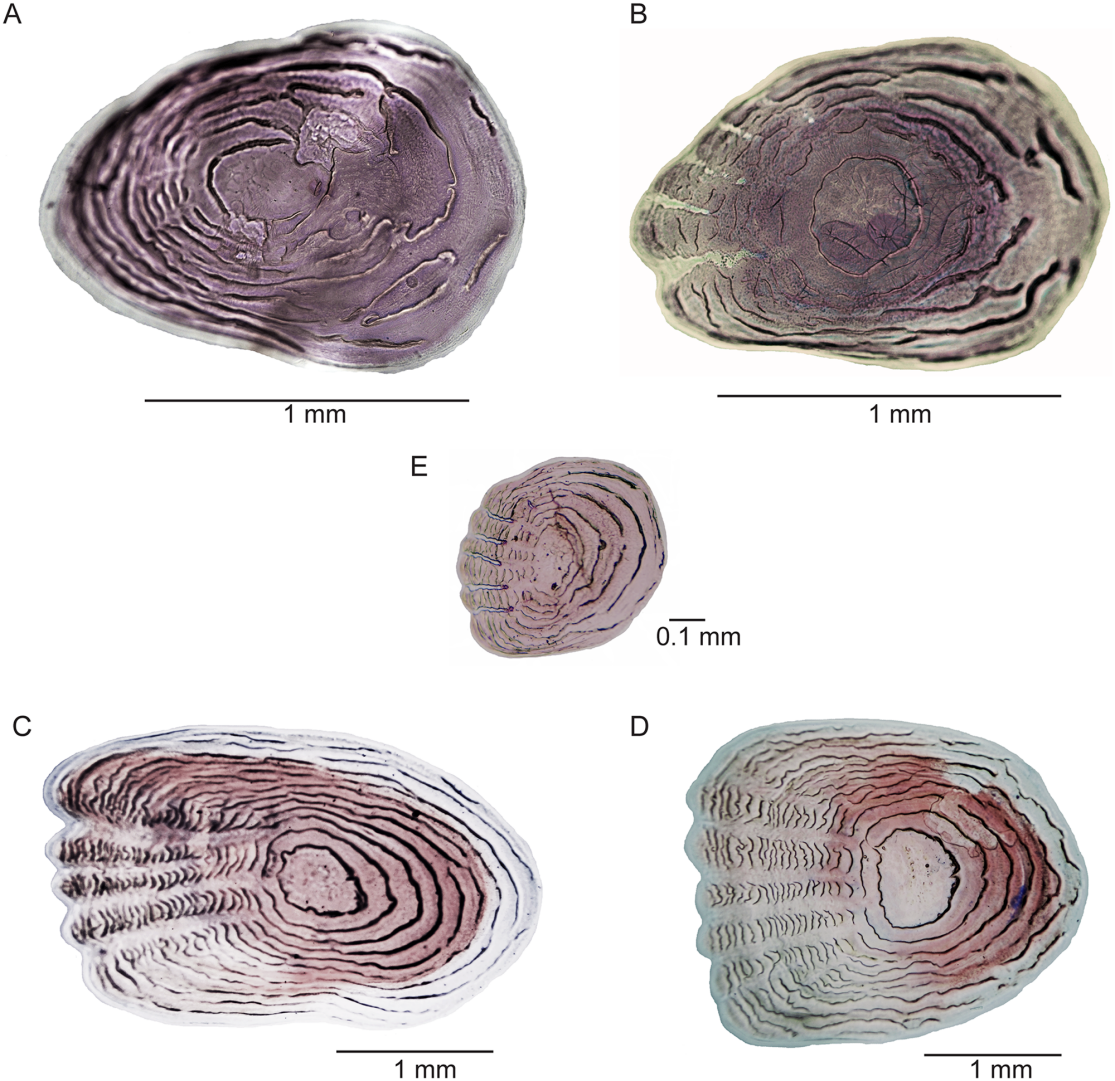
Scales of *Pseudorestias lirimensis* gen. et sp. nov. (female, 61.3 mm SL; KUNHM 41384). **A**, **B**, scales of the flank at the anterior half of the body. **C–E**, scales of the flank at the posterior half of the body.

**Scales** The scales of females and males are thin and delicate. No scutes are present as it the case in some species of *Orestias* where both scales and scutes may be present in one individual. No ctenii are associated with the scales as in some species of *Orestias*, but a few serrations can be found (see below). The scales covering the flanks are rounded or oval-shaped and moderately large in females and males (Fig 16); small scales (Fig 16E) are irregularly distributed among the larger scales, and they are not part of well-defined scale rows. There are about 37 or 38 scales along the mid flank. Unlike species of *Orestias* [3] with a median row of scales, paired scales (Fig 12A) are present in the dorsal median region between the posterior border of the cranium and the insertion of the dorsal fin in *Pseudorestias lirimensis*. These scales can be irregularly positioned and leave some spaces between them and other flank scales. They may also be closely together in the largest females. In contrast, large spaces can be observed between those scales in males. [Reduced or absent body squamation was proposed as a synapomorphy of Orestiini (= *Orestias*) by Costa [4]. A reduced body squamation is common to species of *Orestias*, but not a completely naked skin. Thus, the evidence supporting this character is uncertain. Differences in the occurrence of squamation between females and males are frequently observed.]

**Fig 17:**
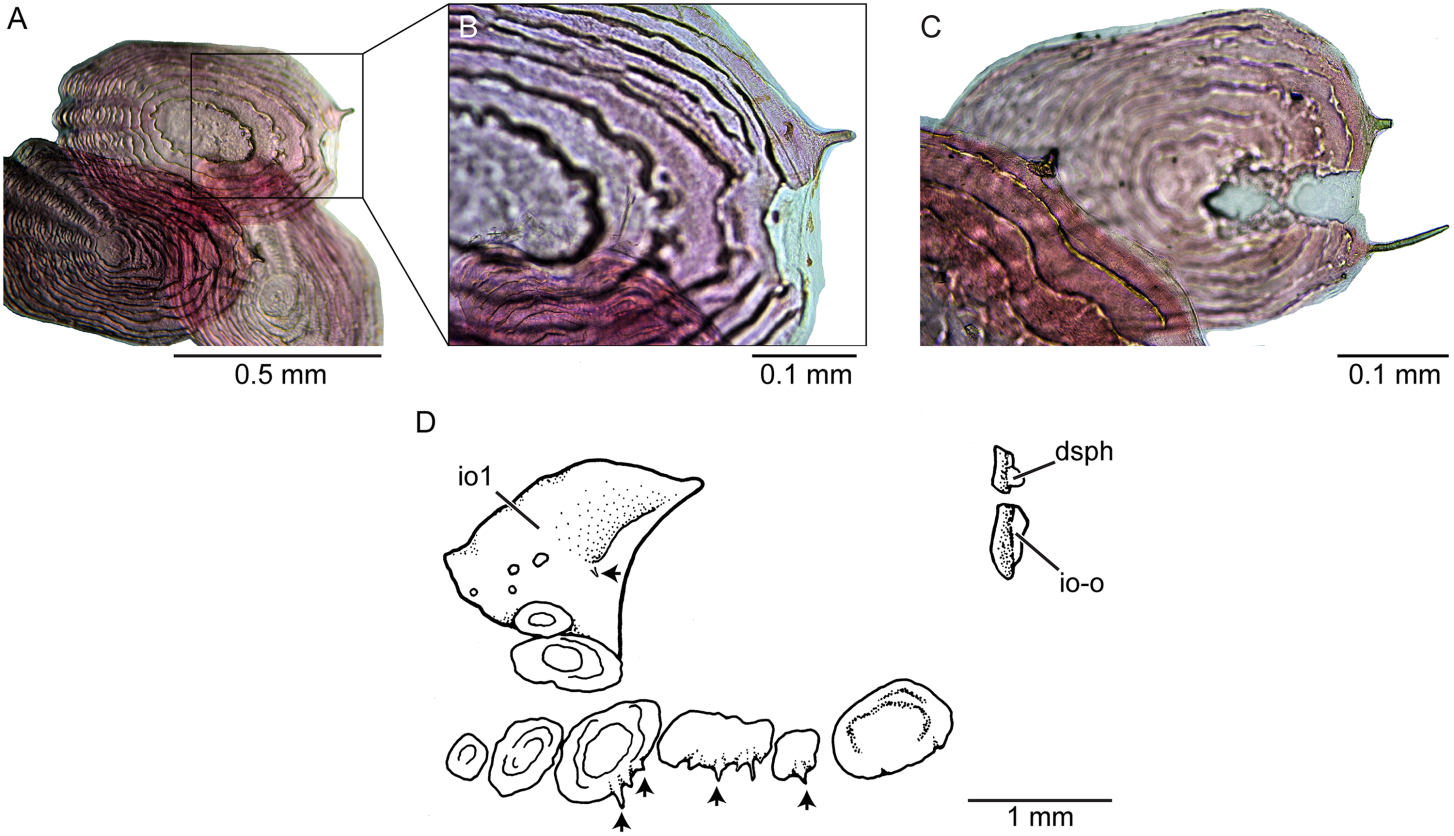
Scales of *Pseudorestias lirimensis* gen. et sp. nov. (male, 30 mm SL; KUNHM 41384). **A–C**, scales of the flank of the posterior half of the body showing a few projections. **D**, orbital bones and scales of the cheek region. Small arrows point to projections of the scales. Abb.: **dsph**, dermosphenotic; **io1**, infraorbital 1; **io-o**, ossicle-like infraorbital bone.

The distribution of scales on the head of females and males differs (Fig 7A–7D). Scales of different sizes and irregularly placed are present on the dorsal aspect of the head in large females. They are concentrated just in the posterior part of the incomplete lyre-like pattern formed by the ethmoid-supraorbital neuromast line (Fig 7A and 7C) or be distributed more extensively (Fig 8A). A few larger scales are placed posteriorly. In contrast, there are two areas with small scales on the dorsal aspect of the head in males: One in the region at the level of the anterior margin of the orbit and another posteriorly (Fig 7D). Large females have larger scales in the cheek region around the orbit and another group of scales in the opercular region (Figs 7A and 8B). Males have small scales irregularly distributed and separated between them (Fig 7B). Serrations (Fig 17B) at the ventral or posteroventral margin of scales in the check region can be observed in males observed under large magnification. Additionally, a few small spines can be present on the lateral surface of the infraorbital 1 in males (Fig 17). Head scales are thin, delicate, and bearing only a few circulii or no circulii at all.

Scales of the flank, placed anteriorly to mid-length of SL, can be oval- or rounded-shaped, with no radii or a few radii in their anterior field (Fig 16A–16B). Only a few, incomplete circulii are present in the posterior field of the scales. Scales placed in the mid-flank of the posterior half of the body are mainly oval or slightly square, with a variable number of radii anteriorly and of circulii in the posterior field (Fig 16D–16E). Spines or projections (not ctenii), irregularly distributed, are also present in cheek scales (Fig 17D) and flank scales (Fig 17A–17C) of males, and occasionally, in some flank scales in females.

**Fig 18:**
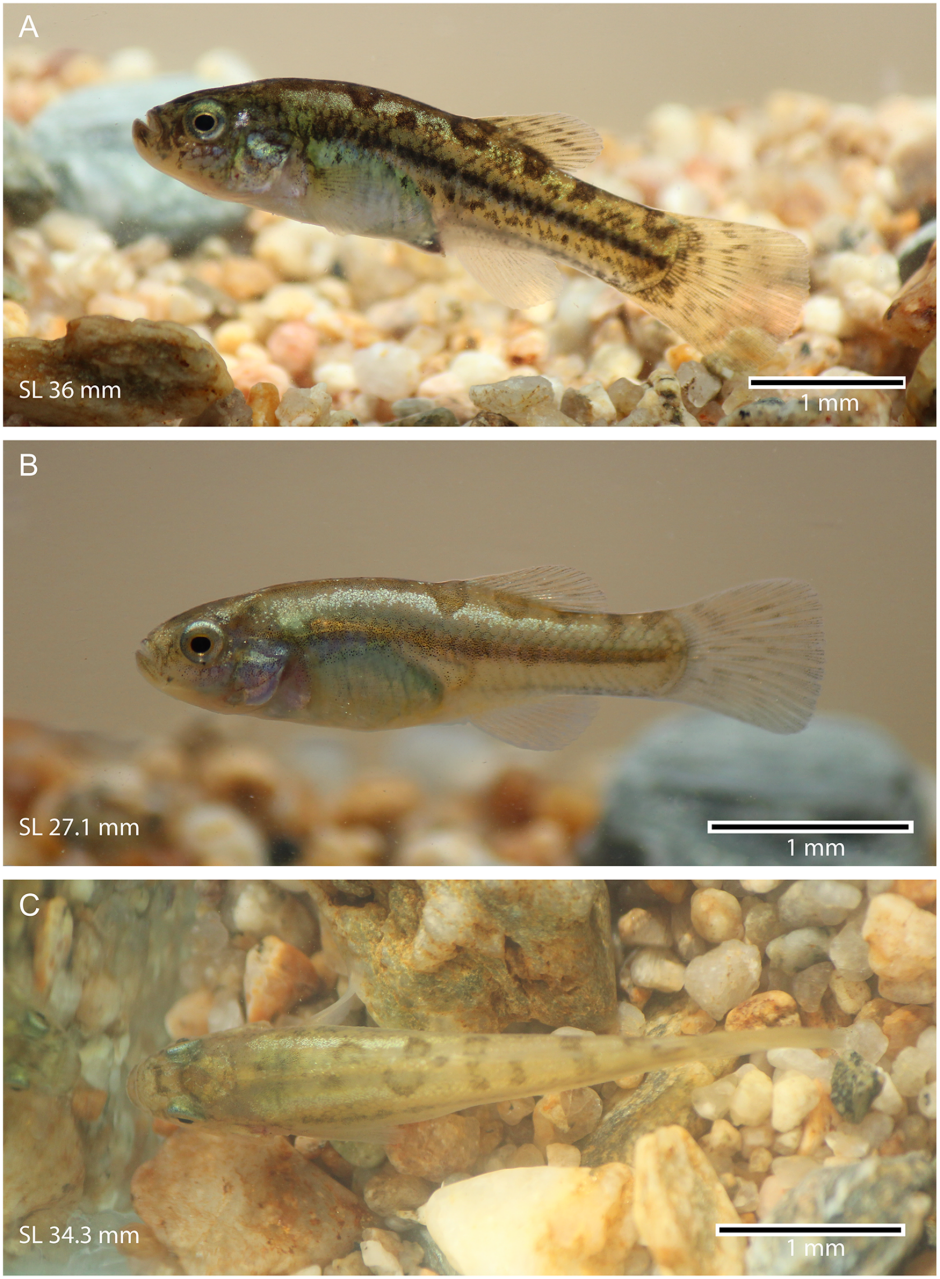
External views of *Pseudorestias lirimensis* gen. et sp. nov. illustrating different coloration patterns. **A**, MHNV 3255, 36 mm SL. **B**, MHNV 3255, 27.1 mm SL. **C**, MHNV 3254, 34.3 mm SL.

**Coloration** The coloration varies strongly between individuals and ontogenetically as is shown in Figs 4 and 18 (representing females). The skin of head and body can produce golden reflections, especially in the opercular region and mid-flank. The protuberant eyes are characterized by a golden cornea surrounding a rounded black pupil. A diffuse blackish band can be present in the mid-flank. It may be more conspicuous in the posterior mid-flank or it is present as a strong dark band accompanied with a row of irregular small spots below it in some individuals. In some individuals, the dorsal region of the body may be orange-brownish. In other individuals, this region may show six to eight irregularly positioned brownish or black spots of different sizes (e.g., Fig 18A–18C). The largest spots are commonly just in front and in the middle-posterior part of the dorsum in front and below the dorsal fin. The ventral region is commonly homogeneously creamy or yellow. The fins are orange-creamy colored with small irregular black spots. Smaller individuals (Fig 4C) are almost transparent with silvery and golden flanks marked by a blackish band along the flank and with large irregularly-shaped spots in the dorsal part of the body. The coloration in males is similar to that illustrated in Fig 4A, but the lateral band is more conspicuous along the whole mid-flank. A second narrow lateral band may be present dorsal to the mid-flank band. No spots have been observed in the dorsal region of the body as those present in females (Figs 4B–4C and 18A–18C).

Female and male specimens fixed in ethanol lose their coloration and become mainly brownish or dark brown-blackish (Fig 8), keeping the blackish band(s) along the flank, and the spots in the case of the females.

**Sexual dimorphism** Males are considerably smaller than females—a feature that it was interpreted as plesiomorphic for killifishes of the genus *Orestia*s [3]. They are usually less than the half of the length of the females. The males are slimmer than females. Sexual dimorphism has also been observed in other features: Females present cephalic neuromast lines lacking in males (see Fig 7). Females have numerous, small, and irregularly shaped scales concentrated in the middle-posterior region of head. Males have few, small, and irregularly distributed scales located between anterior and posterior nostrils and in the postorbital head region (Fig 7). Females have large irregularly shaped scales on the cheek and opercular region. Males have few scales irregularly distributed and separated between them (Fig 7) on the cheek and opercular region as well as on the body. Males have minuscule projections or spines on the ventral or posteroventral margin of scales on head and body (Fig 17). Females may have a few flank scales with a few spines. An uncommon sexual dimorphic character is the presence of an ethmoid rostral cartilage in adult females with no ossified mesethmoid (Fig 5A), whereas adult males have a well-ossified mesethmoid surrounded by a narrow band of cartilage (Fig 5B).

**Fig 19:**
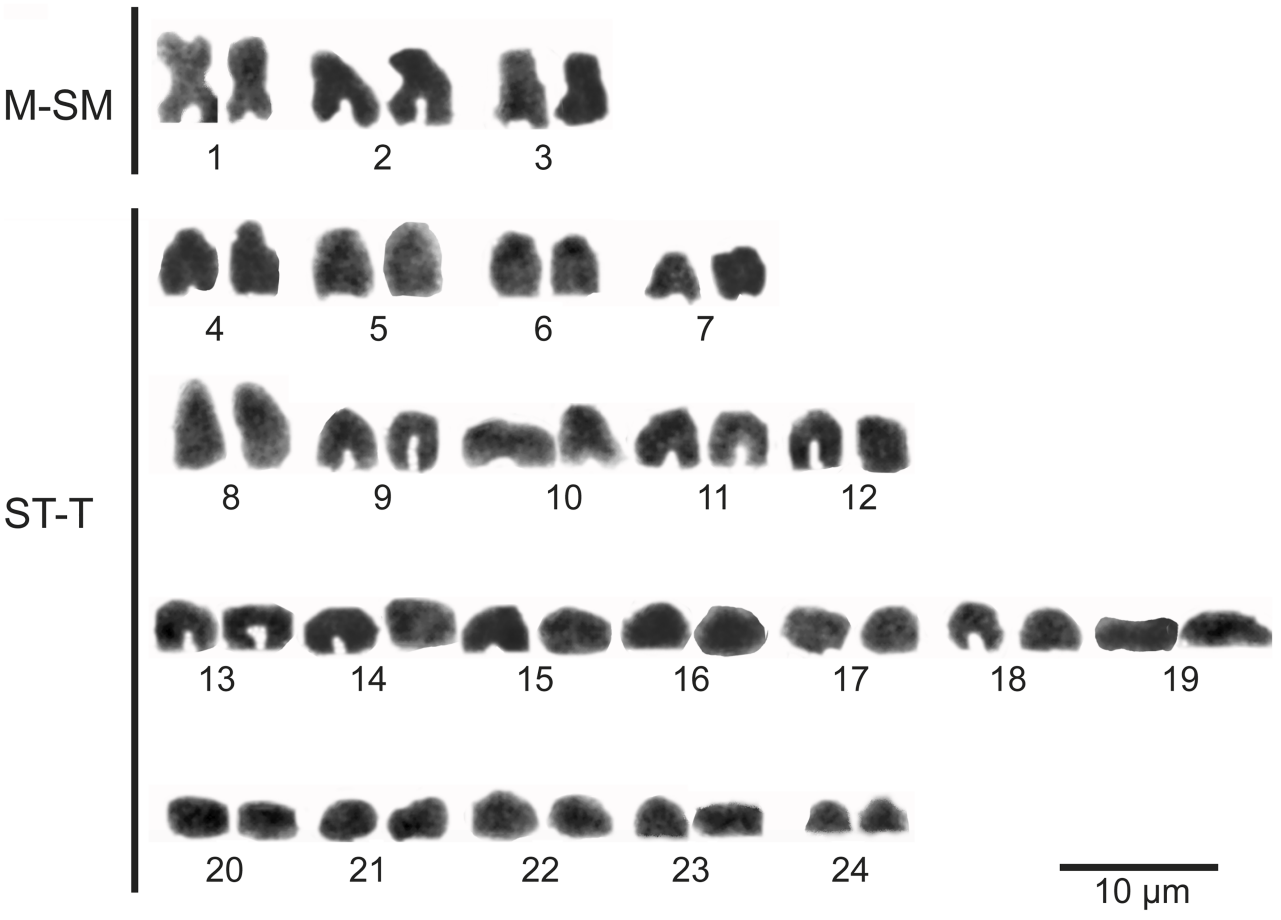
Karyotype of *Pseudorestias lirimensis* gen. et sp. nov. Abb.: **M-SM**, metacentric-submetacentric chromosomes; **ST-T**, subtelocentric-telocentric chromosomes.

**Chromosomes** The diploid number of studied karyotypes is 2n = 48 chromosomes for males and females (Fig 19). That is the most frequent diploid number observed in the genera comprising the family Cyprinodontidae [59]. The karyotype is characterized by three pairs of bi-armed chromosomes and 24 pairs of uni-armed chromosomes. The karyotype formula is: 2M + 4SM + 10ST + 32T, with a chromosome arm number (NF) of 54. This formula is different to that in species of *Orestias* with 48 chromosomes (*O*. *ascotanensis*, *O*. *parinacotensis*, and *O*. cf. *agassi* of Salar de Huasco) described for the Chilean Altiplano [18,23]. Despite having a different chromosomal formula, *Pseudorestias lirimi* has a fundamental arm number (NF) of 54, as in *Orestias*. In addition, it is possible to distinguish one pair of metacentric chromosomes corresponding to the pair 1 (Fig 19) that also is found in species of *Orestias* studied to date [18,23,44]. *Pseudorestias lirimensis* also shares with southern species of *Orestias* four submetacentric chromosomes—a common condition of the species with 48 chromosomes [23]. Morphologically differentiated sexual chromosomes were not found when comparing metaphase plates of males and females. The presence of microcromosomes was not detected as described in some species of *Orestias* [18,37].

## Discussion and final comments

The cyprinodontiform fishes living in the Chancacolla River and Charvinto Creek, near the village of Lirima, are interpreted as a new genus and species. This is based on a set of morphological characters (see Diagnosis) that separate them from members of the genus *Orestias*. Up to now members of the genus *Orestias* have been interpreted as the only known cyprinodontiform of the Altiplano. According to the last publication [4] concerning the phylogenetic position of *Orestias*, the tribe Orestiini would contain only members of *Orestias*. However, such previous interpretation is not supported by the results presented here. *Pseudorestias* is interpreted here as another member of the tribe Orestiini. Among their characters, a few of them deserve further comments.

Among members of Orestiini new usage, the teeth are distributed in a single outer row in both the upper and lower jaws (e.g., [2–4], GA pers. obser.). The teeth may be unicuspid and/or bicuspid in different species of *Orestias* (e.g., [3]). The teeth are unicuspid in *Pseudorestias* gen. nov. The premaxilla and its characteristic dentition and its development have been described ontogenetically in *Aphanius* where more than one row of tricuspid teeth are present [60]. The most external row or labial row has been interpreted as a row of replacement teeth [3,60]. Contrary to *Aphanius*, *Pseudorestias* has a main row of unicuspid teeth and a few replacement teeth, which hardly could be considered as forming an extra row or a replacement row (Fig 9) or an outer row [3]. This so-called replacement row is represented by a few, irregularly placed teeth at different stages of growth, imbedded in the skin forming the upper lip. The presence of replacement teeth (dorsally placed to the oral marginal teeth) is an unusual condition characteristic to certain cyprinodontiforms because the teleostean replacement teeth commonly have a medial or lateral position close to the tooth that they will replace. The ontogenetic development of teeth in *Orestias* and *Pseudorestias* is unknown; thus, an appropriate explanation concerning their dentition is not possible at the present time. Similar outer row of replacement teeth has been mentioned for the lower jaw in *Orestias* [3]. However, in *Pseudorestias* the replacement teeth are irregularly placed medially to the main row of teeth (Figs 9 and 10).

A broadly expanded medial process of the dentary was interpreted as a synapomorphy of Orestiini by Parenti [3], whereas an expanded ventroposterior portion of the dentary was interpreted as a synapomorphy of Cyprinodontidae by Costa [4]. However, an expanded medial process is morphologically not the same as an expanded ventroposterior process of the dentary. *Orestias* and *Pseudorestias* gen. nov. share a broadly expanded medial region of the dentary (not a process) bearing a notch (e.g., Figs 9 and 10). This feature is interpreted here as a synapomorphy of Orestiini new usage. Additionally, the posterior expansion of the Meckel’s cartilage was interpreted as a synapomorphy of Orestiini (= *Orestias*) by Costa [4], despite the fact that the cartilage expands posteriorly in *Aphanius* (see fig 43A in [2]) as it does in *Orestias* (see fig 43B in [2]). In contrast, the posterior expansion of the Meckel’s cartilage was interpreted as a derived character shared by *Orestias* and cyprinodontines by Parenti [2]. *Pseudorestias* has also the Meckel cartilage expanded posteriorly but there are major differences with that of *Orestias* and *Aphanius*. Although similar, the Meckel cartilage of *Aphanius* and *Orestias* loses its anterior part [see fig 43A–B in [2]]. In *Pseudorestias*, the anterior part is kept even in the largest specimens and the expanded posterior part is resorbed (Fig 10B). This feature is interpreted here as an autapomorphy of the new genus.

The infraorbital series of bones is discontinuous in *Orestias* and *Pseudorestias* and is represented by its most anterior bone (the lachrymal or infraorbital 1) and its most posterior bone (the dermosphenotic) and occasionally an ossicle-like posterodorsal infraorbital. While the lachrymal is an expanded, large, flat bone in *Orestias* and *Pseudorestias* (Fig 7), the dermosphenotic is a small ossicle-like, that may be present or not in different species of *Orestias*, but may be present in one or both sides of the body or be completely absent in *Pseudorestias*. The new genus and members of *Orestias* do not have sensory pores opening on the skin surface (e.g., [3,18] and herein). They lack the supra- and infraorbital sensory canals as well as other cephalic canals. Both genera differ in the development of the rostro-ethmoid neuromast lines (especially in the preopercular-mandibular neuromast line that joints its counterpart in *Orestias* [3] but is interrupted in *Pseudorestias*.

Middle dorsal and middle anal radials are cartilaginous in *Orestias* [3,4] (not medial radials as named by Costa [4]). This condition was interpreted as a synapomorphy of Orestiini by Costa [4]. However, *Pseudorestias lirimensis* shows variation on these characters between males and females with males keeping the cartilaginous condition and females, showing different states of ossification along the series of middle dorsal and middle anal radials. It is unclear in the large females whether the middle and posterior radials that are separated in the anterior most pterygiophores become fused caudally. Only future and not yet available studies on an ontogenetic series may clarify this issue.

The presence of thin scales and/or scutes of different sizes in different part of the body are features that have been used to characterize species of *Orestias* in the Altiplano, e.g., the amount of distribution of scales and/or scutes on the dorsum of the head, check, and opercular region, as well as on the body (e.g., [3,18,44]). However, the use of certain patterns of head scales as diagnostic character was questioned under the argument of intraspecific variability for some species of *Orestias* [60]. It is unclear whether some of those differences may represent sexual dimorphism because specimens were not separated by sex. *Pseudorestias lirimensis* exhibits sexual dimorphism in head and body squamation between males and females (e.g., Figs 7, 16 and 17).

A single median row of scales positioned between the occiput and the origin of the dorsal fin is interpreted as a synapomorphy of the species of *Orestias* [3]. In contrast, *Pseudorestias* presents a double row of scales (Fig 12A) in the mid-line between the posterior part of the cranium and the origin of the dorsal fin, a feature interpreted here as an autapomorphy of the genus.

### Sexes and sexual dimorphism, heterochrony, and paedomorphosis

Sexual dimorphism in cyprinodontiforms is a well-known phenomenon, and in *Orestias* is mainly associated with body size [e.g., 2,3,11]. As a rule, females are larger than males and the presence of spinules is associated to one or more unpaired fins. *Pseudorestias lirimensis* also shares such features. The external aspect of an adult male, about the half length of an adult female and slimmer, is similar to a young female (see Fig 4C). Adult males commonly have incomplete squamation in head and body, keeping somehow the early ontogenetic condition of incomplete squamation. In contrast, females have a denser squamation covering head and body. Thus, while females develop a complete squamation, males retain a younger aspect along their lives. There are other features that have not been reported such as the presence versus the absence of neuromast lines in females and males. We do not have any reasonable explanation for such different condition between sexes of *Pseudorestias*. Internally, there appear to be differences in the ossification versus absence of ossification of the dorsal and anal middle and distal radials in females and males and in the presence of a cartilaginous rostral cartilage in adult females with no ossified mesethmoid (Fig 5A), whereas adult males have a well-ossified mesethmoid surrounded by a narrow band of cartilage (Fig 5B). A continuous Meckel’s cartilage in the symphyseal region between both lower jaws in adult *Pseudorestias* is a feature observed in larval stages of different teleosts (GA pers. obser.). This connection is severed in early stages of development. However, its uncommon presence in adult *Pseudorestias* can be interpreted as a paedomorphic condition, which is unknown in *Orestias*. These features as well as the lack and/or reduction of cephalic neuromast lines in males (Fig 7) together with an unossified mesethmoid in females have not been reported previously for other Orestiini. Currently, we do not have explanations for their occurrences in *Pseudorestia*s, but as a consequence of those findings we feel necessary to investigate those characters and others in females and males of *Orestias*. According to our field observations, the number of females versus males of *P*. *lirimensis* is very different in their natural environment. Not only are there more females, but in certain months of the year, males have not been found coexisting with females in the same territory and we have been unable to follow their apparent migrations in the basin. This aspect needs to be investigated further because these observations could be indications of different behaviors between sexes.

### Chromosomes and mitochondrial genome

The chromosome diploid number (48) of the new genus and species is not different from most other cyprinodontiforms [59] and from a few species of *Orestias* such as *O*. *luteus*, *O*. *agassii*, *O*. *parinacotensis*, and *O*. *gloriae* [18,23,44] (see S2 Table). A few exceptions are *O*. *laucaensis* with 50 to 52 chromosomes [18], *O*. *piacotensis* with 52, and *O*. *chungaraensis* with 55 [44]. All those exceptions are from species inhabiting the Lauca Basin of the Chilean Altiplano.

The mitochondrial genome of the new species has been studied [58] as well of that of *Orestias ascotanensis* [61], the only *Orestias* included in the study. Both appear in a sister relationship among other cyprinodontiforms, and exhibit 63 base-pairs (bp) of difference (0.38%) across the mitogenome [58]. *Pseudorestias lirimensis* differs from *O*. *agassi* by 3–5 bp (0.75–1.25%) considering 398 bp of the control region [24,56].

### Ecosystem and its possible history

The Chancacolla River (Fig 1B) is part of the Tarapacá Basin. Its headwaters are located at the western margin of the Andean Altiplano, i.e. the Western Cordillera of the Southern Central Andes. This cordillera corresponds to the late Cenozoic to Recent volcanic arc, where the young, mainly active volcanoes are located along the Chile-Bolivia borderline [62]. Active volcanoes and older volcanic deposits often delineate small, interior drainage basins occupied by saline lakes and salt crusts [20]. The recent volcanic arc comprises the basement of the headwaters of the Chancacolla River. Since Upper Miocene, volcanism has built a substantial positive relief in the upper part and streams of the Chancacolla River [63]. Volcanic activity has been persistent during 10–5 Ma and 4.8–2.6 Ma [63], building a strong water divide from the Titicaca Basin, were its highest summit reaches 5,572 m a.s.l. Although the Chancacolla River might be separated from the Titicaca Basin since the Upper Miocene, a cautionary hypothesis of hydrologic isolation from the Titicaca Basin through these mountains can be established starting at 3 Ma (Gardeweg pers. comm. 2016) and following the formation of a series of stratovolcanoes raised 1500–1800 m above the baseline height [64].

The old, remnant freshwater systems of the Chilean Altiplano to present maintain small populations of cyprinodontiforms and trichomycterid catfishes. Only *Orestias* have been cited for the region. The finding of *Pseudorestias* gen. nov. was unexpected but now it opens the need to revise the most southern populations that have been interpreted as *Orestias* without questioning such assignment. The climatic events which have influenced the development of the freshwater systems from humid periods to long present dry ones have developed smaller lakes, rivers, and wetlands (bofedales) of different salinity levels. The southern freshwater systems located from 17° to 22° S presently have a negative water balance. Together with high salinity soil and volcanic activity, this has incremented the water salinity [36,38,39]. These ecological characteristics and lacking of connection among freshwater systems would have enhanced morphological and physiological adaptations of the fishes and created reproductive isolation among disconnected aquatic systems [34,65–67]. This is the case with *Pseudorestias lirimensis*. Recent studies on *Orestias* living in Ascotán Salar (see S2 Text) suggest that some populations located in the farthest springs of the saltpan are starting to differentiate through genetic drift, generating strong phylogeographic patterns that can be associated with new evolutionary lineages [68].

### Conservation status

According to the criteria of UICN [69] *Pseudorestias lirimensis* gen. et sp. nov. should be categorized as critically endangered species. Its extent of occurrences is estimated to be less than 100 km^2^, and known to exist in a single location (*sensu* [69]), the Chancacolla River and Charvinto Creek near Lirima village. Any water extraction, either superficial or underground that affects the water availability in the Chancacolla River, its streams, peatbogs, and other wetlands associated should be strongly avoided.

## Supporting information

S1 Text List of species of the tribe Orestiini, new usage of the Chilean Altiplano, with map illustrating their geographical distribution. (DOCX)

S2 Text Terms of use of Open Topography. The landscapes used in Fig 1A and 1B are from Open Topography, whereas the geographic position of the Lirima village and rivers are from one of the authors (CQR).
(DOCX)

S1 Fig Diagram of *Pseudorestias lirimensis* gen. et sp. now. Illustrating how the body measurements were taken.
(DOCX)

S1 Table Raw measurements of *Pseudorestias lirimensis* gen. et sp. now. (XLSX)

S2 Table Meristic and karyotype characteristics of *Pseudorestias lirimensis* gen. et sp. nov. compared with species of *Orestias* inhabiting the Chilean Altiplano. (DOCX)
