# Supplementary material for: Morphological and taxonomic descriptions of a new genus and species of killifishes (Teleostei: Cyprinodontiformes) from the high Andes of northern Chile
Source: PLoS One. 2017 Aug 8;12(8):e0181989. doi: 10.1371/journal.pone.0181989 (PMC5549709; doi:10.1371/journal.pone.0181989)
Supplement: S1 Text — (DOCX) [file pone.0181989.s001.docx]

**S2 Text. List of species of the tribe Orestiini, new usage of the Chilean Altiplano, with map illustrating their geographical distribution**

The Tribe Orestiini is represented by eight species in the Chilean Altiplano. These are: *Orestias ascotanensis* Parenti, 1984 [1], *Orestias chungaraensis* Vila and Pinto, 1986 [2], *Orestias gloriae* Vila et al., 2011 [3], *Orestias laucaensis* Arratia, 1982 [4], *O. parinacotensis* Arratia, 1982 [4], and *O. piacotensis* Vila, 2006 [5]. *Pseudorestias lirimensis* gen. et sp. nov. is another taxon apparently unique to the Altiplano. All of these are endemic species. Additionally, *Orestias agassii* Valenciennes, 1846 [6]— with type locality is Corocoro River in San Antonio de Esquilaches, Peru—known from many localities in the Altiplano of Peru and Bolivia [1] has also been cited (but not studied) for the Chilean Altiplano.

Among those species, the ones with the most northern distribution (Fig 1) are *Orestias parinacotensis*, *O. laucaensis*, and *O. piacotensis*, all of then living in the Lauca Basin and its wetlands (bofedales). The most southern species is *O. ascotanensis* living in the freshwaters surrounding the Ascotan Saltpan.

**References**

1. Parenti LR. A taxonomic revision of the Andean killifish genus *Orestias* (Cyprinodontiformes, Cyprinodontidaae). Bull Am Museum Nat Hist. 1984;178: 107–214.

2. Vila I, Pinto M. A new species of killifish (Pisces, Cyprinodontidae) from the Chilean Altiplano. Rev d’Hydrobiologie Trop. 1986;19: 233–239.

3. Vila I, Scott S, Méndez MA, Valenzuela F, Iturra P, Poulin E. *Orestias gloriae*, a new species of cyprinodontid fish from saltpan spring of the southern high Andes (Teleostei: Cyprinodontidae). Ichthyol Explor Freshwaters. 2011;22: 345–353.

4. Arratia G. Peces del altiplano de Chile. In: Veloso A, Bustos-Obregón E, editors. El Ambiente Natural y las Poblaciones Humanas de los Andes del Norte Grande de Chile (Arica, Lat 18°28’S) UNESCO, MAB-6, vol 1, La Vegetación y los Vertebrados Inferiores de los Pisos Altitudinales entre Arica y Lago Chungará. Montevideo, Uruguay: Oficina Regional de Ciencia y Tecnología de la UNESCO para América Latina y el Caribe; 1982. pp. 93–133.

5. Vila I. A New Species of Killifish in the Genus *Orestias* (Teleostei: Cyprinodontidae) from the Southern High Andes, Chile. Copeia. 2006;3: 472–477. doi:10.1643/0045-8511(2006)2006[472:ANSOKI]2.0.CO;2

6. Cuvier G, Valenciennes A. Histoire Naturelle des Poissons. Paris; 1846.

**
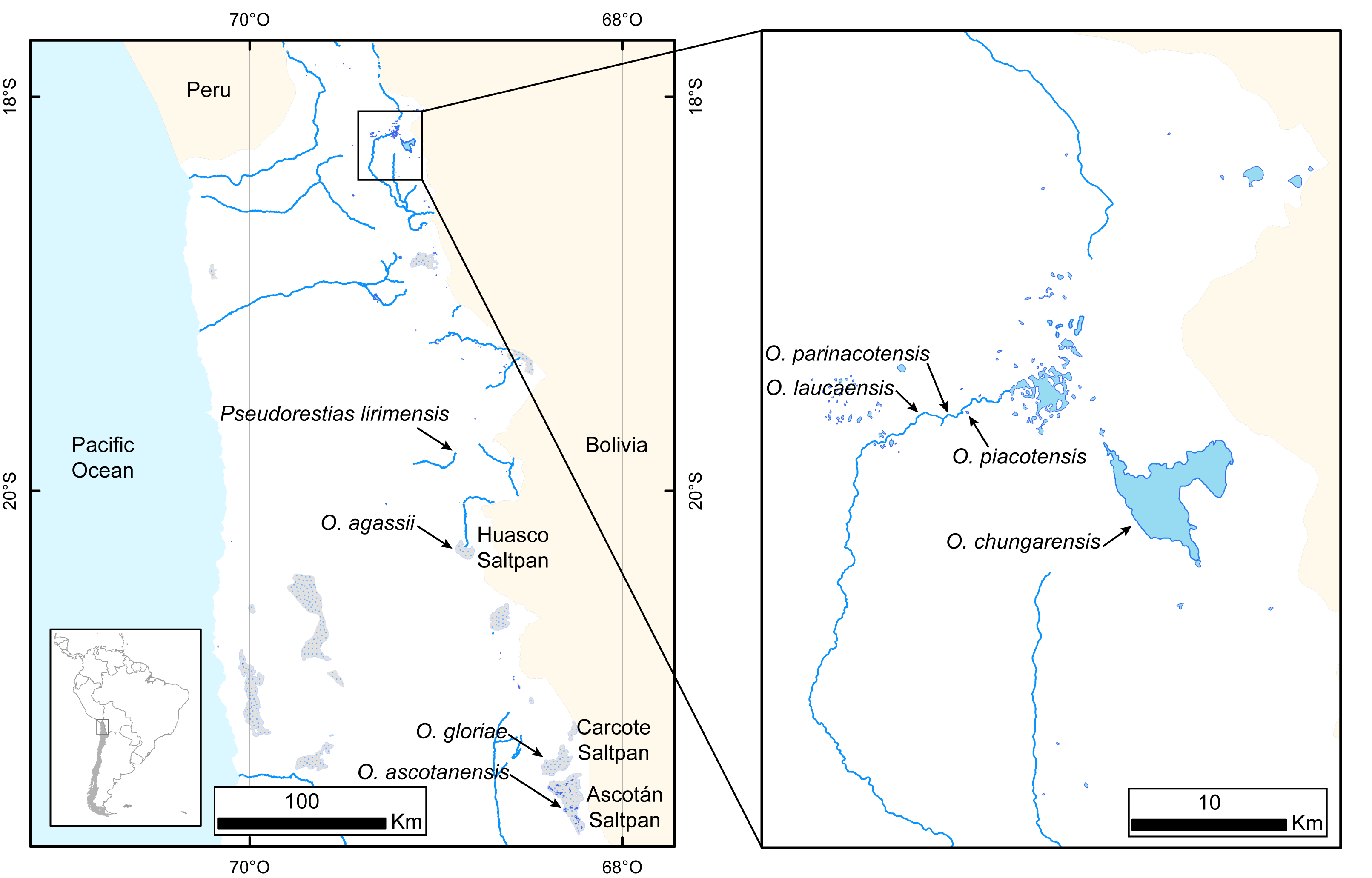
**

**Fig 1. Geographic distribution of members of the Tribe Orestiini in the Chilean Altiplano.**
