# Supplementary material for: Morphological and taxonomic descriptions of a new genus and species of killifishes (Teleostei: Cyprinodontiformes) from the high Andes of northern Chile
Source: PLoS One. 2017 Aug 8;12(8):e0181989. doi: 10.1371/journal.pone.0181989 (PMC5549709; doi:10.1371/journal.pone.0181989)
Supplement: S2 Text — The landscapes used in Fig 1A and 1B are from Open Topography, whereas the geographic position of the Lirima village and rivers are from one of the authors (CQR). (DOCX) [file pone.0181989.s002.docx]

**S2 Text. Terms of use of Open Topography. The landscapes used in Fig 1A, B are from Open Topography, whereas the geographic position of the Lirima village and identification of rivers are from one of the authors (CQR).**

[
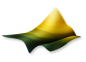
](http://www.opentopography.org/)

[**OpenTopography**](http://www.opentopography.org/)

*High-Resolution Topography Data and Tools*

- [**HOME**](http://www.opentopography.org/)
- [**ABOUT**](http://www.opentopography.org/about)
- [**DATA**](http://opentopo.sdsc.edu/datasets)
- [**TOOLS**](http://opentopo.sdsc.edu/tools/listTools)
- [**LEARN**](http://www.opentopography.org/learn)
- [**COMMUNITY**](http://www.opentopography.org/community)

**Terms of Use**

Data obtained from the OpenTopography Facility are free of all copyright restrictions and made fully and freely available for both non-commercial and commercial use. Users of the data should attribute the source of the data as well as OpenTopography for providing access to those data. Instructions for acknowledging OpenTopography and the data we host can be found on the Citing OpenTopography and Hosted Data page.

Text can only be reprinted with permission, with attribution, and without the right to manipulate or change its content. To request permission, please contact [info@opentopography.org](mailto:info@opentopography.org).

All other articles and text on the OpenTopography site are copyrighted. Permission to use these materials may be requested by contacting [info@opentopography.org](mailto:info@opentopography.org).

**Disclaimer**

OpenTopography makes no guarantee of the quality, reliability, usability, availability, or suitability of any OpenTopography data for any particular purpose. OpenTopography data should not be used for any life-critical functions. Users assume all risks and liabilities, direct or indirect, associated with any use of OpenTopography data.

**Acknowledgements**

Data Access Acknowledgement: Fig 1A-B is based on [data, processing] services provided by the OpenTopography Facility with support from the National Science Foundation under NSF Award Numbers 1226353 & 1225810.

Dataset Acknowledgement:

<http://www2.jpl.nasa.gov/srtm/srtmBibliography.html>

<https://doi.org/10.5069/G9445JDF>

Dataset Name: Shuttle Radar Topography Mission (SRTM GL1) Global 30m(SRTM_GL1)
