# Supplementary material for: Morphological and taxonomic descriptions of a new genus and species of killifishes (Teleostei: Cyprinodontiformes) from the high Andes of northern Chile
Source: PLoS One. 2017 Aug 8;12(8):e0181989. doi: 10.1371/journal.pone.0181989 (PMC5549709; doi:10.1371/journal.pone.0181989)
Supplement: S1 Fig — Illustrating how the body measurements were taken. (DOCX) [file pone.0181989.s003.docx]

**S1 Fig. Diagram of *Pseudorestias lirimensis* gen. et sp. nov. Illustrating body measurements.** Abb.: **AFB**, anal fin base length; **AFH**, anal fin depth; **BD**, body depth; **CPD**, caudal fin peduncle depth; **CPL**, caudal fin peduncle length; **DFB**, dorsal fin base length; **DFH**, dorsal fin depth; **ED**, eye diameter; **HD**, head depth; **HL**, head length; **PAL**, preanal length; **PDL**, predorsal length; **PFL**, pectoral fin length; **POL**, preorbital length; **SL**, standard length; **TL,** total length.

**
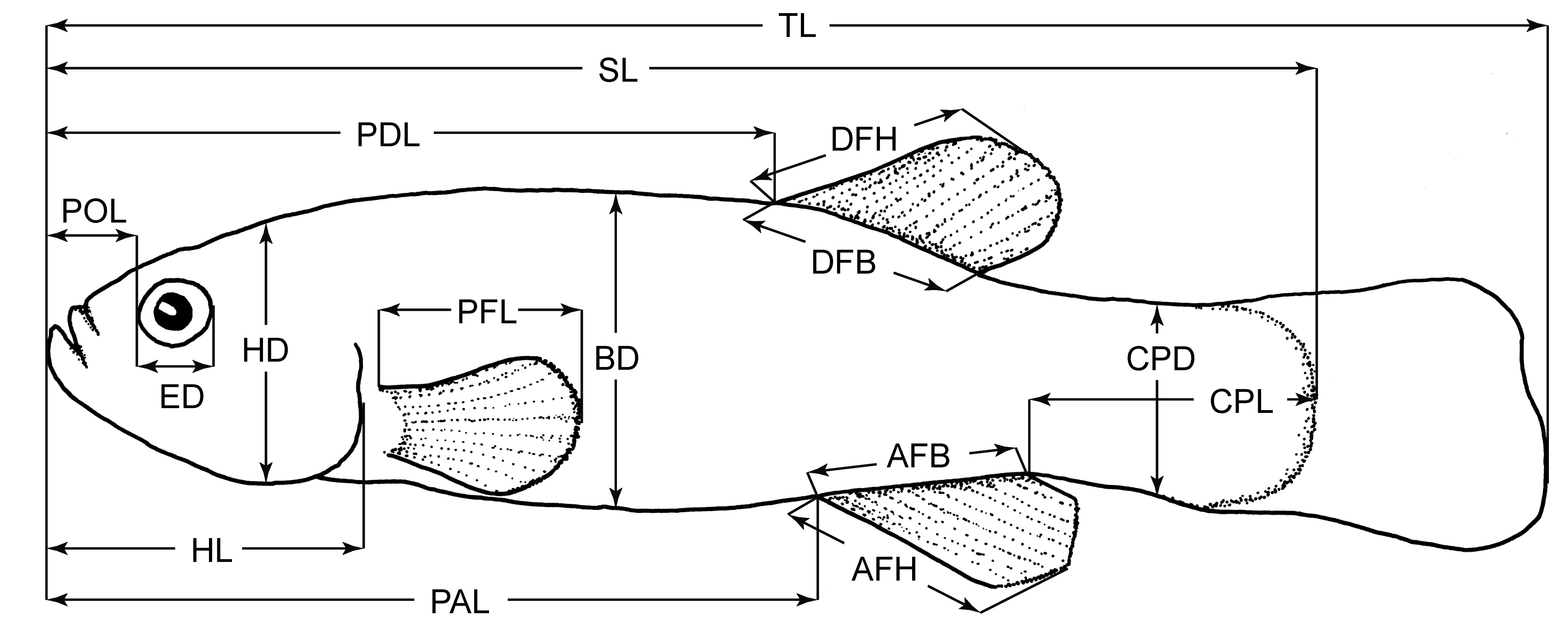
**
