# Supplementary material for: Morphological and taxonomic descriptions of a new genus and species of killifishes (Teleostei: Cyprinodontiformes) from the high Andes of northern Chile
Source: PLoS One. 2017 Aug 8;12(8):e0181989. doi: 10.1371/journal.pone.0181989 (PMC5549709; doi:10.1371/journal.pone.0181989)
Supplement: S2 Table — (DOCX) [file pone.0181989.s005.docx]

- **S2 Table. Meristic and karyotype characteristics of *Pseudorestias lirimensis* gen. et sp. nov. compared with species of *Orestias* inhabiting the Chilean Altiplano.**

| Species | Maximum | Scales | Rays in | Rays in | Rays in | Rays in | Vertebrae | Karyotype |
| --- | --- | --- | --- | --- | --- | --- | --- | --- |
|  | Length (mm) | in mid-flank | pectoral fin | dorsal fin | anal fin | caudal fin |  | 2n |
| *Pseudorestias lirimensis* gen. and sp. nov. | ~75 | 30-35 | 14-17 | 11-16 | 12-15 | 40-41 | 34-36 | 48 |
|  |  |  |  |  |  |  |  |  |
| *Orestias agassii* [1] | ~54 | 30-37 | 14-20 | 11-16 | 11-16 | 22-26 | 31-35 | 48 |
| *O. ascotanensis* [2] | ~75 | 29-34 | 17-19 | 12-15 | 12-14 | 21-35 | 31-32 | 48 |
| *O. chungarensis* [3] | ~82 | 33-35 | 16-18 | 11-14 | 11-14 | 29-32 | 31-32 | 55 |
| *O. gloriae* [4] | ~59 | 31-37 | 16-19 | 14-15 | 13-16 | 23-27 | 29-31 | 48 |
| *O. laucaensis* [5] | ~90 | 33-40 | 15-16 | 14-15 | 14-15 | 36-41 | 33 | 50-52 |
| *O. parinacotensis* [5] | ~85 | 30-37 | 16-20 | 14-17 | 14-15 | 35-39 | 31-32 | 48 |
| *O. piacotensis* [6] | ~72 | 30-34 | 14-16 | 12-14 | 12-14 | 21-27 | 32-34 | 52 |

**References**

1. Cuvier G, Valenciennes A. Histoire Naturelle des Poissons. Paris; 1846.

2. Parenti LR. A taxonomic revision of the Andean killifish genus *Orestias* (Cyprinodontiformes, Cyprinodontidaae). Bull Am Museum Nat Hist. 1984;178: 107–214.

3. Vila I, Pinto M. A new species of killifish (Pisces, Cyprinodontidae) from the Chilean Altiplano. Rev d’Hydrobiologie Trop. 1986;19: 233–239.

4. Vila I, Scott S, Méndez MA, Valenzuela F, Iturra P, Poulin E. *Orestias gloriae*, a new species of cyprinodontid fish from saltpan spring of the southern high Andes (Teleostei: Cyprinodontidae). Ichthyol Explor Freshwaters. 2011;22: 345–353.

5. Arratia G. Peces del altiplano de Chile. In: Veloso A, Bustos-Obregón E, editors. El Ambiente Natural y las Poblaciones Humanas de los Andes del Norte Grande de Chile (Arica, Lat 18°28’S) UNESCO, MAB-6, vol 1, La Vegetación y los Vertebrados Inferiores de los Pisos Altitudinales entre Arica y Lago Chungará. Montevideo, Uruguay: Oficina Regional de Ciencia y Tecnología de la UNESCO para América Latina y el Caribe; 1982. pp. 93–133.

6. Vila I. A New Species of Killifish in the Genus *Orestias* (Teleostei: Cyprinodontidae) from the Southern High Andes, Chile. Copeia. 2006;3: 472–477. doi:10.1643/0045-8511(2006)2006[472:ANSOKI]2.0.CO;2
